# Comparative characterization of volatile flavor profiles and quality attributes of laboratory-prepared and commercial braised chicken feet using a multi-analytical approach

**DOI:** 10.1016/j.fochx.2026.104683

**Published:** 2026-10-26

**Authors:** Zijing Zhang, Haoyang Xie, Yongqiang An, Zihao Meng, Mengjie Li, Longfei Wang, Lishui Chen

**Affiliations:** CGCC Key Laboratory of Nutrition and Health for Snacks, Henan Provincial Intelligent Manufacturing Engineering Technology Research Center for Meat Products, Food Laboratory of Zhongyuan, Luohe 462300, China

**Keywords:** Candidate aroma-active compounds, Braised chicken feet, Flavor chemistry, GC-IMS, OPLS-DA, ROAV, Sensory evaluation

## Abstract

Braised chicken feet are a key leisure snack, yet commercial flavor chemistry differences remain underexplored. We analyzed six samples (one laboratory-prepared, five commercial) using HS-GC-IMS, electronic nose, sensory evaluation, and multivariate statistics. 81 volatiles were identified across 10 classes, among which 12 candidate aroma-active compounds (ROAV ≥1) were the main contributors to aroma differentiation. OPLS-DA clearly separated the six samples (R^2^Y = 0.996, Q^2^ = 0.991). 2-Acetyl-1-pyrroline, 2-ethyl-4-methylthiazole, and linalool showed the highest VIP scores. Notably, nonanal showed a nominally significant positive correlation with flavor (*r* = 0.83, *P* < 0.05), suggesting that lipid oxidation-derived aldehydes likely contribute to the common aroma background. The laboratory-prepared sample received the highest scores for flavor, mouthfeel, and taste, although only taste differed significantly from all commercial samples. Flavor formation was proposed to arise from lipid oxidation, the Maillard reaction, and spice-derived terpenes. These findings offer insights for flavor standardization and product development.

## Highlights

• A flavor wheel of braised chicken feet was built from 81 volatiles in 10 classes.
• 12 candidate aroma-active compounds (ROAV ≥1) discriminated the six samples.
• OPLS-DA clearly separated the six samples (R^2^Y = 0.996, Q^2^ = 0.991).
• 2-Acetyl-1-pyrroline, 2-ethyl-4-methylthiazole, and linalool ranked highest by VIP.

## Graphical abstract

**Figure:**
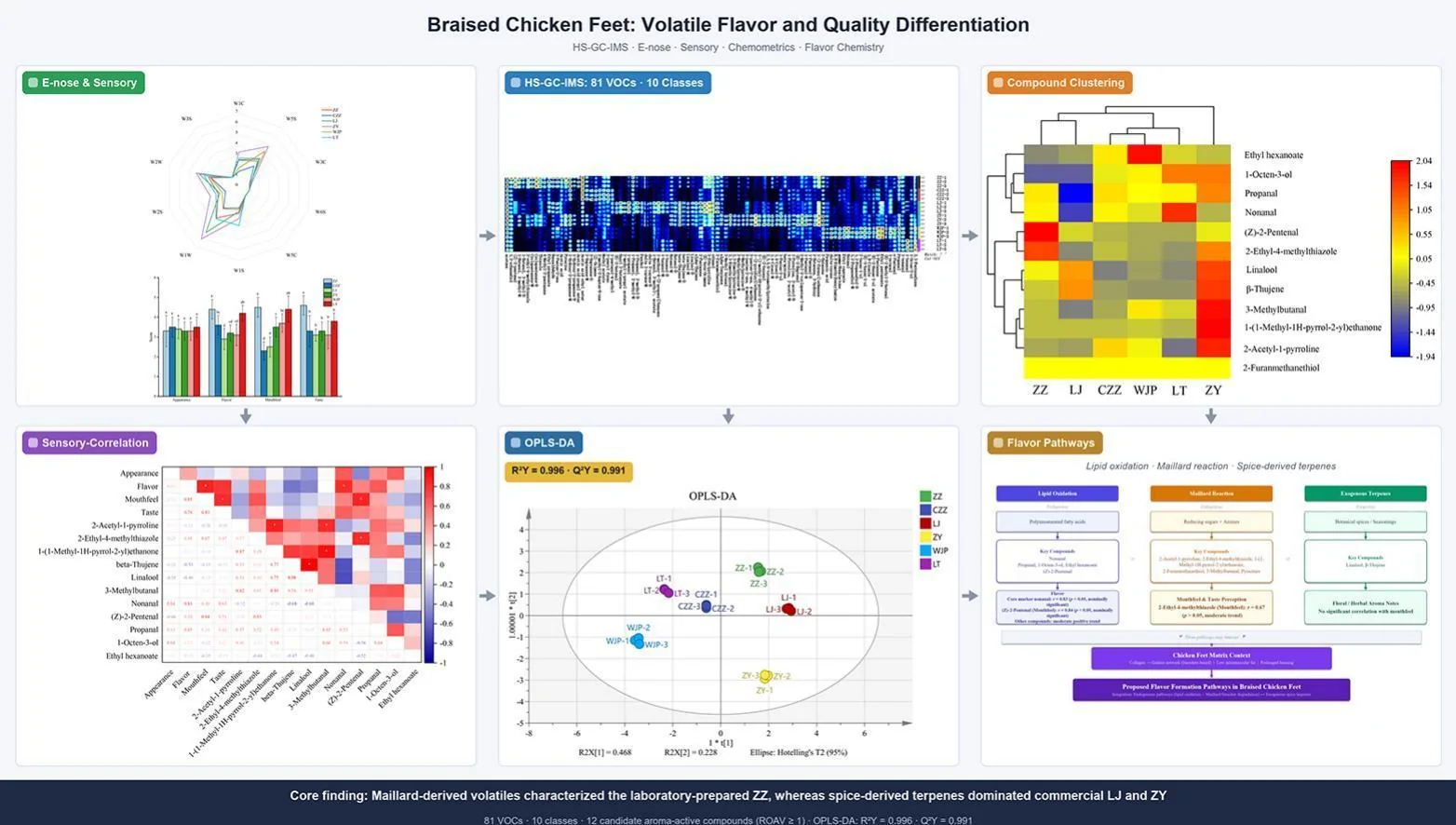

## 1 Introduction

Braised chicken feet seasoned with traditional Chinese spices have long been an integral part of China's culinary culture. In recent years, their commercialization has accelerated alongside the rapid expansion of the leisure snack industry, with China's snack food market reaching 105.2 billion USD in 2025 (Grand View Research, 2026). Among braised poultry products, chicken feet have emerged as a particularly dynamic category, largely because of their high collagen content and the distinctive chewy texture it imparts (Santana et al., 2020; Usman et al., 2024). Several brands now compete in this market, each differentiated by proprietary spice blends and processing methods that directly influence the final aroma profile. Flavor is widely recognized as the most important factor (Fu et al., 2022; Liu et al., 2023) driving consumer acceptance and repeat purchase of braised meat products. However, the chemical basis of flavor differences among commercial brands has received little systematic investigation. Addressing this knowledge gap requires instrumentation-based characterization of flavor-related compounds. The aroma of braised chicken is highly complex, arising from several thermally induced reactions occurring simultaneously, including Maillard reactions (Bassam et al., 2022; Shakoor et al., 2022), lipid oxidation and degradation (Fu et al., 2022; Geng et al., 2023), and heat-induced release of terpenoids and phenolic compounds from added spices, which together generate a wide spectrum of volatile compounds that contribute to the overall aroma profile (Wang, Wang, et al., 2023; Zhan et al., 2022). Headspace gas chromatography-ion mobility spectrometry (HS-GC-IMS) has emerged as a powerful analytical tool (Lv et al., 2024; Ma et al., 2024) for volatile fingerprinting (Yang et al., 2023) in food matrices because it operates under atmospheric pressure, needs minimal sample preparation, and offers high sensitivity for detecting trace-level volatile organic compounds (VOCs). When paired with principal component analysis (PCA) and orthogonal partial least squares discriminant analysis (OPLS-DA), HS-GC-IMS provides a robust approach for sample discrimination and aroma marker identification. In addition, the relative odor activity value (ROAV) further refines aroma evaluation (Liang et al., 2023; Zhang, Li, et al., 2025) by normalizing the odor activity value (OAV) of each compound to that of the compound with the highest OAV, thereby facilitating the identification of the key contributors to aroma perception without requiring the quantification of every individual odorant.

Beyond volatile fingerprints, several physicochemical properties closely associated with product quality and flavor release also warrant investigation in connective tissue-rich matrices such as chicken feet. The pH influences collagen gel characteristics (Rezabeigi et al., 2023), water distribution, and the partitioning of flavor compounds between the food matrix and the headspace. Malondialdehyde (MDA), a secondary marker of lipid oxidation (Jiao et al., 2020), is closely associated with warmed-over flavor (Chen, Zhang, et al., 2024; Zhang, Li, et al., 2025) and overall flavor deterioration in meat products (Bleicher et al., 2022). Although chicken feet contain negligible intramuscular fat, lipid oxidation in subcutaneous and connective tissues can still contribute to aroma development. Surface color, particularly the characteristic reddish-brown hue of braised products, serves as an important visual quality attribute for consumers and indirectly reflects the extent of Maillard browning (Cheng et al., 2022; Yao et al., 2025). A critical gap in the existing literature is that most studies investigating the flavor chemistry of braised chicken (Yao, Wu, et al., 2024) have focused on muscle-dominant cuts (Sun et al., 2023; Sun, Yu, et al., 2024), such as breast, thigh, or whole carcass, where flavor development is primarily governed by intramuscular lipid oxidation and protein degradation. Chicken feet represent a fundamentally different substrate. They consist predominantly of skin and connective tissue, with a high collagen content and negligible intramuscular fat (Esmaeili-Kaliji et al., 2025; Zourazema et al., 2025). This unique composition alters the thermal reaction pathways, affecting volatile compound partitioning and shifting the primary source of flavor compounds from intramuscular lipid oxidation to the penetration of exogenous spice components, together with the mild thermal degradation of skin and connective tissue components. Consequently, flavor differentiation in chicken feet is likely to be more subtle and less predictable than that observed in conventional muscle-based products, and findings from previous studies may not be directly applicable (Esmaeili-Kaliji et al., 2025). Although the quality attributes and cooking behavior of chicken feet have been characterized under various cooking conditions (Yu et al., 2024), no study to date has systematically integrated volatile, physicochemical, and sensory analyses to compare commercially available braised chicken feet.

The present study was designed to address this knowledge gap. Six braised chicken feet samples were examined, including five commercial brands (Caizhizhai, Liaoji Bangbangji, Ziyan Baiweiji, Wangjiapo, and Lintai) and one laboratory-prepared sample produced under controlled formulation conditions as a reference for evaluating matrix effects. The analytical approach integrated HS-GC-IMS for volatile fingerprinting, ROAV analysis to identify candidate aroma-active compounds, PCA and OPLS-DA for pattern recognition, electronic nose analysis for overall aroma discrimination, sensory evaluation for assessing sensory attributes, and physicochemical measurements (pH, MDA, and color) for matrix-associated quality characterization. The objectives were to: (1) characterize volatile fingerprints that differentiate the six samples; (2) identify the candidate aroma-active compounds responsible for sample-specific aroma profiles; (3) investigate the correlations between multi-platform instrumental measurements and sensory attributes; and (4) generate evidence-based guidance for flavor optimization. From a fundamental perspective, this study provides a systematic multi-platform characterization of the flavor chemistry of high-collagen, connective-tissue-based braised products, addressing a longstanding gap in muscle-centric poultry research. From an application perspective, the results provide practical benchmarks for flavor standardization and product differentiation within this rapidly expanding market.

## 2 Materials and methods

### 2.1 Materials

Five commercially available brands of braised chicken feet within their shelf life were selected based on market research: Caizhizhai Suzhou-style soy-braised chicken feet (CZZ, Dalian Bangchuidao Food Co., Ltd., Dalian, China), Liaoji Bashu-style spicy braised chicken feet (LJ, Chengdu Liaoji Bashu Food Co., Ltd., Chengdu, China), Ziyan Baiweiji spiced braised chicken feet (ZY, Hubei Ziyan Food Co., Ltd., Wuhan, China), Wangjiapo Cantonese-style salt-braised chicken feet (WJP, Guangdong Wangjiapo Food Co., Ltd., Chaozhou, China), and Lintai Cantonese-style salt-baked chicken feet (LT, Jiaoling Lintai Longevity Food Co., Ltd., Meizhou, China). A laboratory-prepared sample (ZZ) was produced according to the procedure described in Section 2.2. n-Ketones (C4-C9), including 2-butanone, 2-pentanone, 2-hexanone, 2-heptanone, 2-octanone, and 2-nonanone (≥ 99% purity, Aladdin Biochemical Technology Co., Ltd., Shanghai, China), were used as external standards (Ma et al., 2024) for retention index calibration. Nitrogen (≥ 99.999%) was purchased from Shanghai Yuanye Biochemical Technology Co., Ltd. (Shanghai, China). Ultrapure water (18.2 MΩ·cm at 25 °C, Milli-Q, Merck Millipore, Billerica, MA, USA) was employed throughout the experiment.

All five commercial samples were purchased online in July 2025 and shipped by standard express courier in their original, unopened vacuum packages; no ice packs or other temperature-control measures were used during transit. Upon arrival, the packages were kept in their original vacuum-sealed state and stored at 4 °C until analysis. ZZ was prepared in July 2025 and vacuum-packed as described in Section 2.2. The commercial samples were produced in the same month (as declared on the package label) and carried declared shelf lives of 60–270 d; all were analyzed well within these limits. The in-house physicochemical (pH, MDA, and color), electronic-nose, and sensory analyses were completed within a three-day window approximately one week after purchase, whereas volatile profiling by GC-IMS was performed separately by a commercial analytical service provider (Hanon Advanced Technology Group Co., Ltd., Jinan, China), as described in Section 2.8.2. The transit duration and transit temperature were not recorded or controlled and are accordingly acknowledged as potential sources of variation among the commercial samples.

### 2.2 Sample preparation

To prepare the laboratory-prepared sample (ZZ) following the method described by Yao et al. (Yao et al., 2022) with modifications, chicken feet (10 kg) were tumbled with maltose and cooking wine for 20 min to facilitate deodorization and sugar coating. The feet were then fried in oil at 180 °C for 5 min until a golden-yellow color developed on the skin, followed by immediate immersion in ice water (0–4 °C) for 30 min. This rapid cooling step induced the characteristic “tiger-skin” wrinkled texture on the surface. In parallel, star anise (60 g), green and red Sichuan peppercorns (30 g each), Angelica dahurica (30 g), cinnamon bark (30 g), bay leaves (12 g), clove (7.5 g), Amomum villosum (10 g), tsaoko (15 g), nutmeg (15 g), and fennel seeds (15 g) were combined with 30 L of water and simmered over low heat for 1 h to produce an aromatic spice infusion. The spice bag was removed, and seasoning ingredients, including salt (1125 g), light soy sauce (150 g), yeast extract (60 g), chicken bone broth paste (600 g), spicy chicken flavor paste (1200 g), ethyl maltol (30 g), monosodium glutamate (60 g), and white granulated sugar (1200 g), were incorporated. The mixture was then brought to a boil to prepare the final braising broth. The pre-fried chicken feet (10 kg) were added to the boiling marinade and simmered over low heat for 30 min to permit penetration of the spices and seasonings into the skin and connective tissues. After cooking, the heat was turned off, and the samples were allowed to steep in the residual heat for an additional 30 min. The braised samples were vacuum-packed and sterilized at 121 °C for 20 min to ensure commercial sterility.

### 2.3 Sensory evaluation

Sensory evaluation was conducted with minor modifications in accordance with the general principles described by Tura et al. (Tura et al., 2024) and standard sensory evaluation practices. All evaluations were performed in a dedicated sensory evaluation room. A panel of 10 trained assessors (five males and five females, aged 22–40 years) evaluated the samples. All assessors were healthy, non-smoking, free of known taste or olfactory disorders, and regular consumers of braised meat products. Before formal testing, the assessors received two 1-h training sessions to familiarize themselves with the sample type, the sensory descriptors, and the rating scale, and reached consensus on the definitions of each attribute through discussion. Four attributes—appearance, flavor, mouthfeel, and taste—were evaluated using a 5-point descriptive intensity rating scale (1–5, integer scores), with each attribute anchored at three descriptive levels (1–2 = weak, 3–4 = moderate, 5 = strong). Before serving, the vacuum-packed samples were reheated in a 90 °C water bath for 10 min, manually deboned, and cut into uniform pieces of approximately 2 g (approximately 30 g per serving). Each portion was placed in a lidded white ceramic cup, coded with a random three-digit number, and served warm at 60 ± 5 °C under uniform lighting in individual sensory booths. The assessors were blinded to the identity and brand of the samples, and the serving order was randomized among panelists to minimize order effects. Between samples, panelists cleansed their palates with purified water and unsalted soda crackers, with a 2-min rest interval to reduce sensory fatigue.

### 2.4 Determination of pH

The pH was measured in accordance with the National Standard of China (GB 5009.237–2016) (National Health and Family Planning Commission of China, 2016b) with minor modifications. Briefly, 5.0 g of homogenized sample was mixed with 45.0 mL of ultrapure water, homogenized for 2 min, and allowed to stand for 30 min at room temperature. The pH of the supernatant was determined using a calibrated pH meter (Mettler Toledo FE20, Zurich, Switzerland). All measurements were performed in triplicate (*n* = 3).

### 2.5 Determination of MDA

The MDA content was determined in accordance with the National Standard of China (GB 5009.181–2016) (National Health and Family Planning Commission of China, 2016a), with minor modifications. Briefly, 5.0 g of homogenized sample was mixed with 5.0 mL of trichloroacetic acid (TCA, 15% *w*/*v*) and vortexed for 2 min. After centrifugation at 4000 ×*g* for 15 min at 4 °C, the supernatant (2.0 mL) was combined with 2.0 mL of thiobarbituric acid (TBA, 0.02 mol/L). This mixture was heated in a boiling water bath for 20 min, rapidly cooled under running tap water, and the absorbance was measured at 532 nm using a UV–Vis spectrophotometer (Shimadzu UV-2600, Kyoto, Japan). The MDA concentration was calculated using the following equation:

$$\mathrm{MDA}\left(\mathrm{mg}/\mathrm{kg}\right)=\frac{A_{532}\times V\times M}{\varepsilon \times l\times m}$$

where A₅₃₂ represents the absorbance at 532 nm, V represents the total volume (mL), M represents the molar mass of MDA (72.06 g/mol), ε represents the molar extinction coefficient (1.56 × 10^5^ L·mol^−1^·cm^−1^), l represents the optical path length (1.0 cm), and m represents the sample mass (g). All measurements were performed in triplicate (*n* = 3).

### 2.6 Determination of color difference

Color determination was performed following the method described by King et al. (King et al., 2023) with minor modifications. The color parameters of braised chicken feet samples were measured using a CR-400 portable colorimeter (Konica Minolta, Tokyo, Japan). Before each measurement session, the instrument was calibrated using a standard white ceramic tile (calibration values: Y = 93.8, x = 0.3159, y = 0.3323) under controlled ambient conditions (25 ± 1 °C and 50 ± 5% relative humidity).

For each sample, five intact, uniformly sized chicken feet were randomly selected, and excess surface brine was gently blotted off with filter paper. A single measurement was performed on the palm skin region of each foot, which was selected as the most representative area because of its relatively uniform color and minimal variation. The final color value for each sample was calculated as the average of the five individual measurements. Outlier data points exceeding 2 standard deviations from the mean were excluded from the final calculation.

Color was expressed using the CIE L*a*b* color system (Yao et al., 2025), where *L*^*⁎*^ represents lightness, ranging from 0 (black) to 100 (white); *a*^*⁎*^ represents the red-green coordinate, where negative values denote green and positive values denote red; and *b*^*⁎*^ represents the yellow-blue coordinate, where negative values denote blue and positive values denote yellow. The browning index (BI), which reflects the degree of non-enzymatic browning, was calculated using the following equation:

$$x=\frac{a^{*}+1.75L^{*}}{5.645L^{*}+a^{*}-3.012b^{*}}$$

$$\mathrm{BI}=\frac{100\times \left(x-0.31\right)}{0.17}$$

All measurements were performed in quintuplicate (*n* = 5).

### 2.7 Electronic nose test

Electronic nose analysis was performed following the methods described by Chen et al. and Munekata et al. (Chen et al., 2025; Munekata et al., 2023). The PEN3 system (AIRSENSE Analytics GmbH, Schwerin, Germany), equipped with 10 metal oxide semiconductor sensors, was used to detect volatile compounds, including sulfur-containing compounds, nitrogen-containing compounds, alcohols, and aromatic compounds (Cho & Moazzem, 2022; Tan & Xu, 2020). The instrument was preheated for 30 min before each measurement session. During analysis, the sensors were cleaned with purified air for 120 s until the response values approached 1.0000. Sample detection was performed at a flow rate of 300 mL/min with a signal acquisition time of 80 s, and the corresponding sensor response values were recorded and stored. Because the sensor signals reached a stable equilibrium after 40–60 s of detection, the conductivity values at 60 s were selected for subsequent analyses to ensure signal stability and data accuracy.

For each sample, approximately 3.0 g of homogenized braised chicken feet was placed in a 250 mL Erlenmeyer flask, which was then sealed tightly with parafilm. The flask was incubated in an 80 °C water bath for 40 min to allow headspace equilibrium. Preliminary optimization confirmed that 40 min was sufficient for volatile headspace equilibration. Each sample was prepared in triplicate and analyzed three times to minimize experimental errors.

### 2.8 GC-IMS analysis

#### 2.8.1 Sample preparation

Each sample was deboned, and approximately 4.0 g of the sample was weighed into a 20 mL headspace vial (Agilent Technologies, Palo Alto, CA, USA). The vials were incubated at 80 °C for 15 min to facilitate the release of volatile compounds. All samples were analyzed in triplicate (*n* = 3).

#### 2.8.2 GC-IMS analysis

Volatile compounds were analyzed using a GC-IMS system consisting of an Agilent 490 gas chromatograph (Agilent Technologies, Palo Alto, CA, USA) coupled with a FlavorSpec® IMS instrument (Gesellschaft für Analytische Sensorsysteme mbH, Dortmund, Germany). Samples were introduced using an automated headspace sampler (CTC PAL-3, CTC Analytics AG, Switzerland). GC-IMS measurements were performed by a commercial analytical service provider (Hanon Advanced Technology Group Co., Ltd., Jinan, China) following the manufacturer's standard protocol and established HS-GC-IMS procedures (Li et al., 2022); the resulting raw data were processed in-house.

Headspace injection conditions were as follows: incubation temperature, 80 °C; incubation time, 15 min; injection volume, 500 μL; injection mode, splitless; agitation speed, 500 rpm; and needle temperature, 85 °C.

GC conditions: Separation was performed using a DB-WAX capillary column (15 m × 0.53 mm, 1.0 μm film thickness) maintained at 60 °C. High-purity helium (≥ 99.999%) was used as the carrier gas. The flow rate program was as follows: an initial flow rate of 2.0 mL/min was maintained for 2 min, followed by a linear increase to 10.0 mL/min over 8 min, a subsequent increase to 100.0 mL/min over 10 min, and a final hold at 100.0 mL/min for 20 min. The total run time was 40 min. The injector temperature was maintained at 80 °C.

IMS conditions: High-purity nitrogen (≥ 99.999%) was used as the drift gas at a flow rate of 75 mL/min. Volatile compounds were ionized in positive ion mode and transported through a 53 mm drift tube maintained at 45 °C. Retention indices (RI) were calibrated using a mixed standard solution of n-ketones (C4–C9, 10 mg/L each in HPLC-grade methanol (≥ 99.9% purity), Sinopharm Chemical Reagent Beijing Co., Ltd., China) as external standards for retention index calibration only (not for concentration calibration). Compound identification was based on two criteria, RI and drift time (relative to the reactant ion peak, RIP), by comparison with the built-in NIST-based GC-IMS library. Peak areas were used for relative quantification, in which the peak area of each compound was normalized to the total peak area of all identified volatiles. For compounds detected as monomer/dimer (and occasionally trimer) signal pairs, the peak areas of all forms were summed before normalization. All compound identities should be regarded as tentative, being assigned solely on the basis of RI and drift-time matching against the GC-IMS library; definitive confirmation would require GC–MS analysis. Spectral data and volatile fingerprints were generated using the Reporter and Gallery Plot plugins (G.A.S., Germany).

### 2.9 ROAV calculation

The ROAV was calculated according to the method described by Zhang et al. (Zhang, Li, et al., 2025), which is based on the OAV concept (Chen, Zhu, et al., 2024; Liang et al., 2023). The ROAV was calculated using the following equation:

$$\mathrm{ROAV}\approx 100\times \left(\frac{C_{i}}{C_{\max}}\right)\times \left(\frac{T_{\max}}{T_{i}}\right)$$

where Ci and Ti represent the relative peak area and odor threshold of compound i, respectively, and Cmax and Tmax represent the relative peak area and odor threshold of the compound with the highest odor activity value among all detected volatile compounds. Compounds with ROAV ≥1 were regarded as candidate aroma-active compounds, whereas compounds with 0.1 ≤ ROAV <1 were considered aroma modifiers. It should be noted that ROAV ≥1 indicates an estimated odor activity of at least 1% relative to the reference compound and does not necessarily imply OAV ≥ 1; the ROAV values reported here are peak-area-based estimates. In this study, the reference compound was 2-furanmethanethiol, which had the highest odor activity value and was therefore assigned an ROAV of 100. Odor threshold values were obtained from the database compiled by van Gemert (van Gemert, 2011).

### 2.10 Statistical analysis

Data are expressed as mean ± standard deviation (SD). Before conducting one-way ANOVA, data normality was assessed using the Shapiro-Wilk test, and homogeneity of variance was evaluated using Levene's test. Of note, each sample was represented by a single production batch, and the replicate measurements (*n* = 3 for GC-IMS, pH, MDA, and electronic-nose analyses; *n* = 5 for color parameters) were technical/analytical replicates of that batch rather than independent production lots. Accordingly, the present study constitutes an exploratory single-batch comparison, and the results should be interpreted with caution and not extrapolated to brand-level inference. Batch-to-batch replication was not assessed; this single-batch design is therefore acknowledged as a limitation, and replication across independent production batches is identified as a direction for future work.

GC-IMS data were processed using Laboratory Analytical Viewer (LAV, G.A.S., Germany) with built-in plugins, together with the GC-IMS library search system (NIST and IMS databases). Volatile fingerprints and topographic plots were generated using the Reporter and Gallery Plot plugins (G.A.S., Germany).

Multivariate statistical analyses, including PCA, OPLS-DA, and variable importance in projection (VIP) analysis, were performed using SIMCA 14.1 software (Umetrics, Umeå, Sweden). PCA and OPLS-DA were performed on the relative peak areas of the 12 compounds with ROAV ≥1, with variables scaled to unit variance (UV). For OPLS-DA modeling, the class variable was sample (six classes), and the model was validated using sevenfold cross-validation.Variables with VIP values >1.0 were considered potential markers for sample discrimination. A supplementary PCA and OPLS-DA based on all 81 detected compounds is provided in the Supplementary Material (Fig. S1–S4).

One-way analysis of variance (ANOVA), followed by Tukey's honestly significant difference (HSD) post hoc test, was performed using IBM SPSS Statistics 26.0 (IBM Corp., Armonk, NY, USA) to identify significant differences among groups (*P* < 0.05). Pearson correlation analysis was also performed using the same software. Pearson correlations were based on six sample means (effective *n* = 6); with *df* = 4, the threshold for significance at *P* < 0.05 is *r* ≈ 0.81. Given this exploratory sample size, correlations with *r* ≈ 0.6 are reported as trends only, and no formal correction for multiple comparisons was applied. All graphs, except those generated for multivariate statistical analyses, were plotted using Origin 2025 software (OriginLab Corp., Northampton, MA, USA).

## 3 Results and discussion

### 3.1 Physicochemical and sensory analysis

#### 3.1.1 Physicochemical properties and color parameters

The pH value of the six samples ranged from the lowest in LT to the highest in ZY, with all samples falling within a near-neutral to slightly alkaline range (Table 1). ZZ, LJ, and ZY exhibited higher pH values than CZZ, WJP, and LT. This difference may be attributable to a greater release of basic amino acids from protein hydrolysis during braising (Khalid et al., 2023). Because pH directly influences water-holding capacity, collagen gelation (Rezabeigi et al., 2023; Santana et al., 2020), and the ionization state of flavor-active compounds, these inter-sample differences may contribute to perceptible variations in both texture and flavor.

**Table 1:** Physicochemical properties and color parameters of braised chicken feet samples.

Sample	pH	MDA (mg/kg)	*L*^⁎^	*a*^⁎^	*b*^⁎^	BI
ZZ	7.39 ± 0.00^b^	0.17 ± 0.01^e^	35.12 ± 2.46^d^	17.33 ± 1.65^b^	18.95 ± 2.96^c^	109.89 ± 11.83^cd^
CZZ	7.02 ± 0.01^c^	0.43 ± 0.02^d^	45.22 ± 0.69^c^	24.48 ± 2.79^a^	29.04 ± 3.76^b^	135.04 ± 19.97^bc^
LJ	7.39 ± 0.01^b^	0.56 ± 0.01^b^	35.74 ± 1.40^d^	12.67 ± 1.76^c^	20.85 ± 3.82^c^	109.95 ± 20.07^cd^
ZY	7.42 ± 0.01^a^	0.14 ± 0.01^f^	35.64 ± 1.23^d^	14.28 ± 1.39^bc^	18.34 ± 1.76^c^	99.10 ± 6.52^d^
WJP	7.07 ± 0.07^c^	1.76 ± 0.06^a^	50.71 ± 3.02^b^	12.13 ± 3.72^c^	46.47 ± 4.01^a^	191.94 ± 11.48^a^
LT	6.73 ± 0.01^d^	0.49 ± 0.01^c^	61.05 ± 1.46^a^	5.38 ± 1.59^d^	49.06 ± 1.98^a^	144.00 ± 6.49^b^

Sample codes: ZZ, laboratory-prepared sample; CZZ, Caizhizhai; LJ, Liaoji Bangbangji; ZY, Ziyan Baiweiji; WJP, Wangjiapo; LT, Lintai. Data are expressed as mean ± SD (*n* = 3 for pH and MDA; *n* = 5 for color parameters). Different superscript lowercase letters within the same column indicate significant differences (*P* < 0.05) according to Tukey's HSD test.

MDA, a widely used indicator of lipid peroxidation (Chen, Zhang, et al., 2024; Zhang, Dai, et al., 2025), differed significantly among the six samples (Table 1), ranging from the lowest value in ZY to the highest in WJP. ZY and ZZ exhibited the lowest MDA levels, indicating greater oxidative stability, whereas WJP showed an elevated MDA level, suggesting more extensive lipid oxidation (Al-Dalali et al., 2022). LJ, LT, and CZZ exhibited intermediate MDA levels. The significantly high MDA content observed in WJP may be associated with greater oxidation of subcutaneous and inter-connective adipose tissues, potentially reflecting differences in processing intensity or antioxidant protection (Yu, Wang, et al., 2021). Lipid oxidation products such as MDA (Bao et al., 2025; Ji et al., 2024) can react with amino groups to form Schiff bases and contribute to off-flavor development (Ning et al., 2025; Wang, Zhang, et al., 2023). Therefore, the significant variation in MDA levels among the six samples suggests that oxidative stability is an important factor influencing the quality of commercially available braised chicken feet. These findings further underscore the importance of antioxidant strategies, whether through formulation, thermal processing, or packaging, in preserving sensory quality and extending shelf stability.

All color parameters differed significantly among the six samples. LT exhibited the highest *L*^⁎^ and *b*^⁎^ values, whereas ZZ, ZY, and LJ exhibited lower *L*^⁎^ values; CZZ and WJP showed intermediate values. The *a*^⁎^ value of CZZ was significantly higher than that of the other samples (*P* < 0.05). For the browning index (BI), WJP and LT exhibited higher values, whereas ZZ, LJ, and ZY did not differ significantly (*P* > 0.05). The elevated *b*^⁎^ and BI values observed in WJP and LT may reflect more extensive non-enzymatic browning (Cheng et al., 2022; Yao et al., 2025). In contrast, the high *a*^⁎^ value of CZZ is consistent with a more pronounced reddish hue, likely conferred by dark-colored braising ingredients. Collectively, these colorimetric patterns not only reflect variations in thermal processing and formulation but also serve as practical indicators of visual quality, with appropriate browning and reddish-brown hues generally associated with stronger sensory characteristics in braised meat products (Sun, Yu, et al., 2024).

#### 3.1.2 Sensory evaluation

The sensory evaluation results are presented in Fig. 1. Appearance scores showed a relatively narrow range among the six samples, and one-way ANOVA revealed no significant differences for this attribute (*P* > 0.05). In contrast, significant differences were observed among samples for flavor, mouthfeel, and taste attributes (*P* < 0.05). ZZ and LT received the highest scores for both flavor and mouthfeel, with no significant differences between them for either attribute. LJ showed the lowest flavor score, whereas CZZ and LJ received the lowest mouthfeel scores. Taste exhibited the greatest discriminatory capacity among the evaluated attributes, with ZZ achieving significantly higher scores than all other samples (*P* < 0.05), while the five commercial samples showed no significant differences among them.

**Fig. 1:**
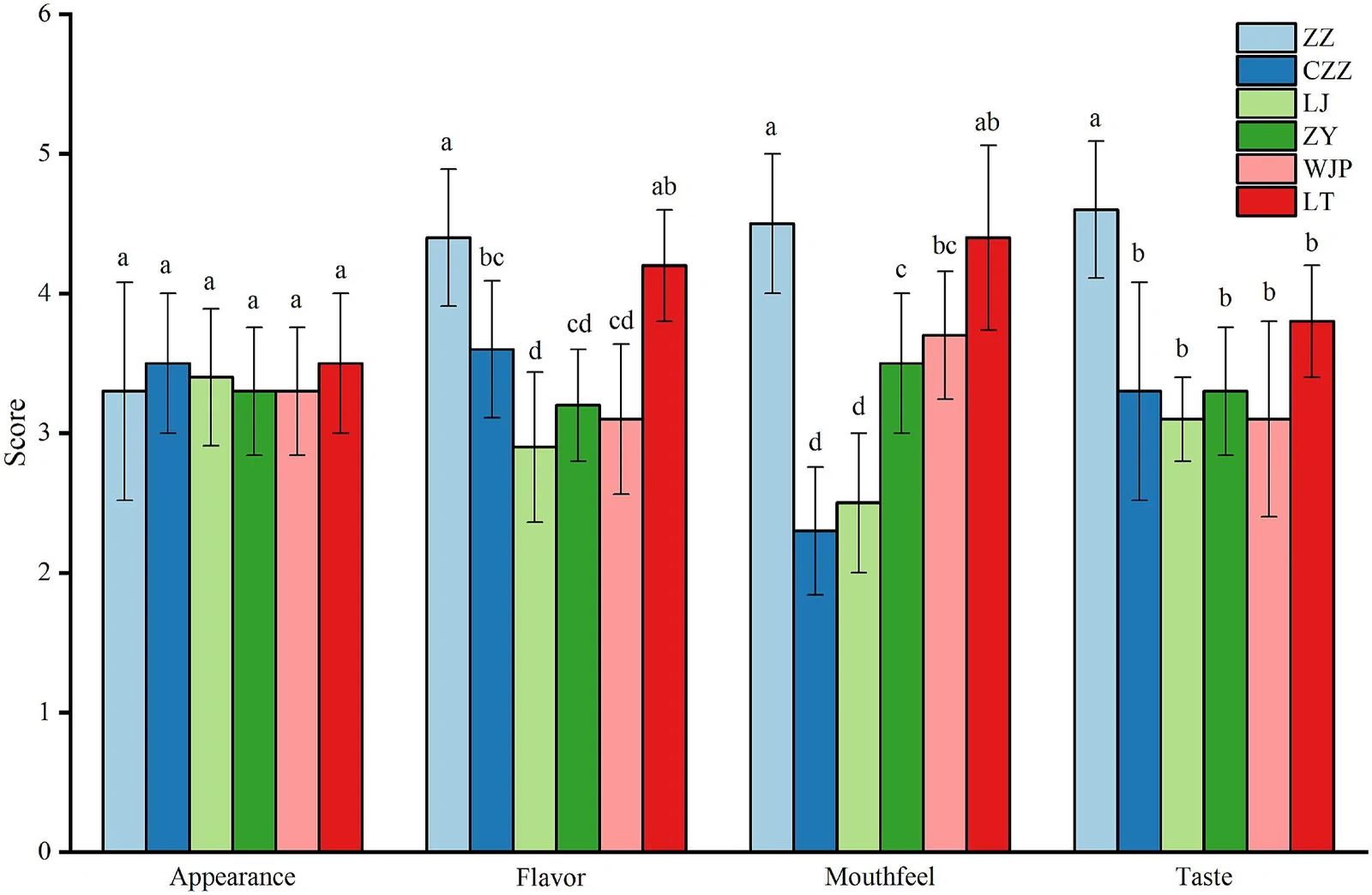
Sensory evaluation scores of braised chicken feet samples. Different lowercase letters above the bars within the same sensory attribute indicate significant differences among samples according to Tukey's HSD test (*P* < 0.05)*.*

### 3.2 Electronic nose analysis

Volatile fingerprints of the six samples were characterized using a PEN3 electronic nose equipped with 10 metal-oxide semiconductor sensors (Cho & Moazzem, 2022; Munekata et al., 2023) (Table 2 and Fig. 2). All ten sensors exhibited significantly different responses among the six samples (*P* < 0.05), indicating that the electronic nose effectively discriminated the volatile profiles of the different samples. Overall, the strongest signals were observed for sensors W1W (sulfur-containing compounds), W5S (nitrogen oxides), W2W (aromatic compounds and organic sulfides), and W1S (broad-range methane), suggesting that sulfur- and nitrogen-containing compounds were the most responsive volatile classes detected by the electronic nose (Chen et al., 2025; Zhou et al., 2022). In contrast, sensors W6S (hydrogen) and W3S (methane and alkanes) exhibited response values close to unity among the samples, reflecting their intrinsically weak signals.

**Table 2:** Electronic nose sensor response values (G/G₀) of braised chicken feet samples.

Sensor Model	Detection Characteristic	ZZ	CZZ	LJ	ZY	WJP	LT
W1C	Aromatic compounds	2.60 ± 0.13^b^	1.50 ± 0.02^d^	2.57 ± 0.12^b^	3.33 ± 0.01^a^	1.83 ± 0.06^c^	2.75 ± 0.05^b^
W5S	Nitrogen oxides	3.26 ± 0.25^b^	2.46 ± 0.35^c^	3.03 ± 0.24^bc^	4.78 ± 0.05^a^	4.23 ± 0.21^a^	3.40 ± 0.14^b^
W3C	Aromatic compounds, ammonia	1.20 ± 0.02^b^	1.06 ± 0.00^d^	1.20 ± 0.01^b^	1.31 ± 0.01^a^	1.10 ± 0.01^c^	1.22 ± 0.01^b^
W6S	Hydrogen	1.05 ± 0.00^a^	1.05 ± 0.00^a^	1.05 ± 0.00^a^	1.04 ± 0.00^b^	1.03 ± 0.00^b^	1.05 ± 0.01^a^
W5C	Alkanes, aromatic compounds	1.05 ± 0.01^b^	1.03 ± 0.00^d^	1.05 ± 0.00^b^	1.07 ± 0.00^a^	1.03 ± 0.00^d^	1.04 ± 0.00^c^
W1S	Broad range, methane	2.52 ± 0.09^c^	2.02 ± 0.07^e^	2.34 ± 0.08^cd^	3.00 ± 0.06^b^	2.15 ± 0.10^de^	3.68 ± 0.09^a^
W1W	Sulfur compounds	3.66 ± 0.48^b^	2.50 ± 0.29^c^	5.37 ± 0.51^a^	6.13 ± 0.16^a^	2.58 ± 0.15^c^	3.75 ± 0.07^b^
W2S	Broad range, alcohols	2.08 ± 0.17^bc^	1.35 ± 0.04^e^	1.93 ± 0.19^cd^	2.87 ± 0.15^a^	1.68 ± 0.04^de^	2.29 ± 0.10^b^
W2W	Aromatic compounds, organic sulfides	3.06 ± 0.23^cd^	2.81 ± 0.30^d^	3.98 ± 0.23^ab^	4.29 ± 0.07^a^	2.86 ± 0.21^d^	3.52 ± 0.02^bc^
W3S	Methane, alkanes	1.14 ± 0.00^ab^	1.12 ± 0.01^ab^	1.14 ± 0.01^ab^	1.12 ± 0.01^b^	1.14 ± 0.01^a^	1.13 ± 0.00^ab^

Data are expressed as mean ± SD (*n* = 3). Different superscript lowercase letters within the same row indicate significant differences among samples according to Tukey's HSD test (*P* < 0.05). G/G₀, sensor response ratio.

**Fig. 2:**
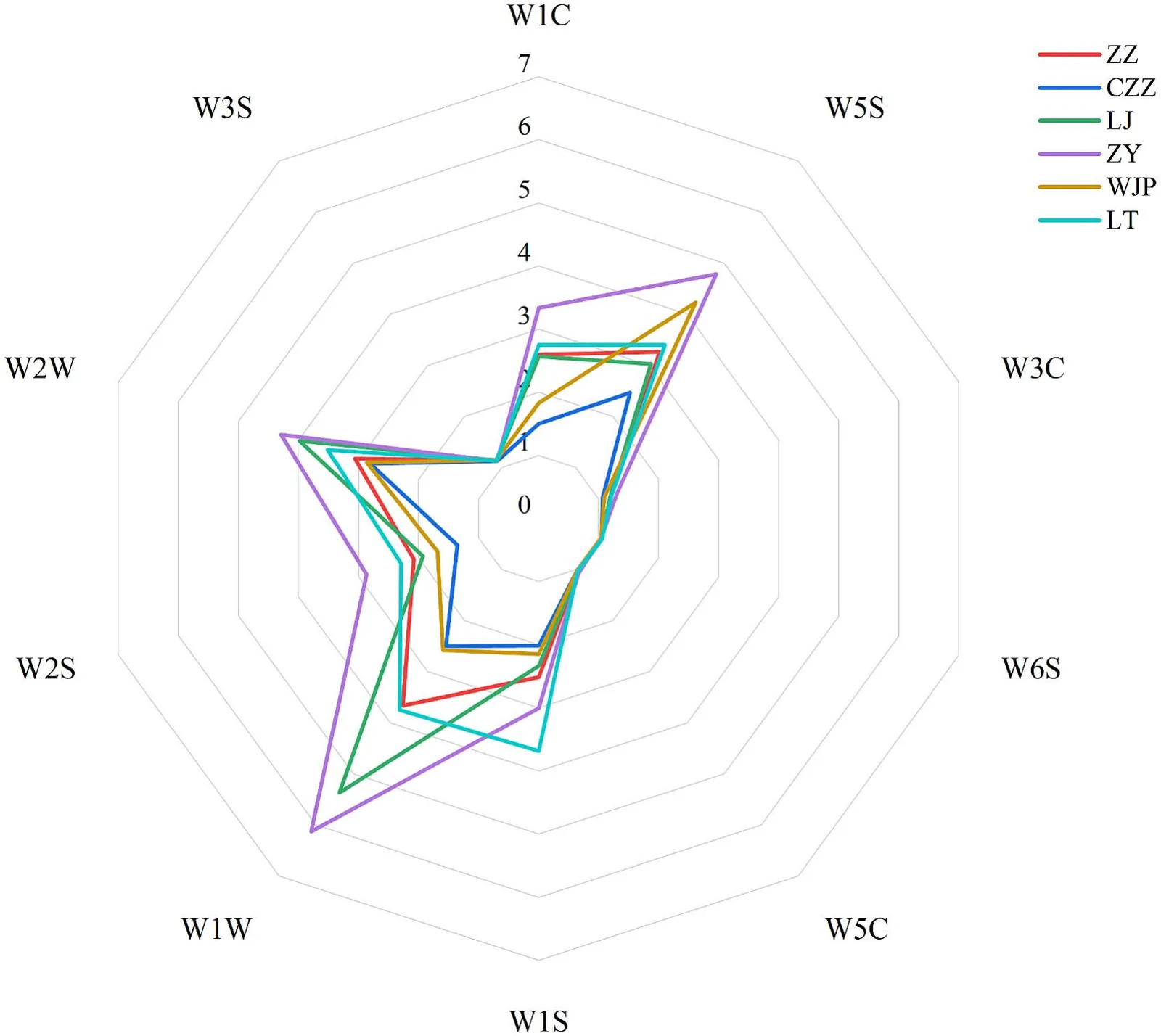
Radar plot of electronic nose sensor responses for braised chicken feet samples.

Among the six samples, ZY exhibited the highest or near-highest (Da et al., 2021; Putri et al., 2023) responses for seven of the ten sensors (W1C, W5S, W3C, W5C, W1W, W2S, and W2W), indicating a relatively complex volatile profile. LJ showed the second-highest responses for the sulfur-sensitive sensors W1W and W2W, following ZY, which is consistent with the relatively high abundance of sulfur-containing volatile aroma compounds subsequently identified by GC-IMS. LT was characterized by the highest response for sensor W1S, whereas ZZ exhibited intermediate responses across most sensors. In contrast, CZZ exhibited the lowest responses for nine sensors, while WJP showed the lowest responses for two sensors, indicating relatively less intense volatile profiles for these two commercial samples. Although the radar plot showed generally similar response patterns among the six samples, differences in response magnitude enabled clear discrimination among the six samples. Collectively, the electronic-nose responses reflected the overall volatile profile of the six samples.

### 3.3 GC-IMS volatile flavor profiling

#### 3.3.1 Overall volatile compound profiles

GC-IMS analysis identified 81 volatile compounds across the six sample batches. These compounds were classified into 10 chemical categories (Ge et al., 2020; Zhou et al., 2022): aldehydes (13), ketones (14), alcohols (14), esters (11), terpenes (12), heterocyclic compounds (8), pyrazines (4), acids (2), other compounds (2), and sulfur-containing compounds (1) (Sun et al., 2023; Sun, Li, et al., 2024). Detailed compound classification is provided in Fig. 3 and Table 3. Fig. 3 presents the GC-IMS spectral data in three complementary formats: a three-dimensional (3D) topographic plot (Fig. 3A), a two-dimensional (2D) contour plot (Fig. 3B), and a gallery fingerprint plot (Fig. 3C). The 3D topographic plot provides an overview of the volatile fingerprint of the six samples. All samples exhibited a dense cluster of common peaks near the baseline at short retention and drift times, representing volatile compounds shared across all six samples. In addition, each sample displayed distinct peak clusters, indicating characteristic volatile compounds. The 2D contour plot further highlighted differences among the samples, particularly between the laboratory-prepared sample and the five commercial samples. Arranged in vertical columns against a common RIP reference line, the laboratory-prepared sample showed a higher density of peaks in the early-eluting region, which was mainly associated with aldehydes and short-chain alcohols. In contrast, the commercial samples showed relatively stronger signals for terpenes and pyrazine-type nitrogen heterocycles at later retention times.

**Fig. 3:**
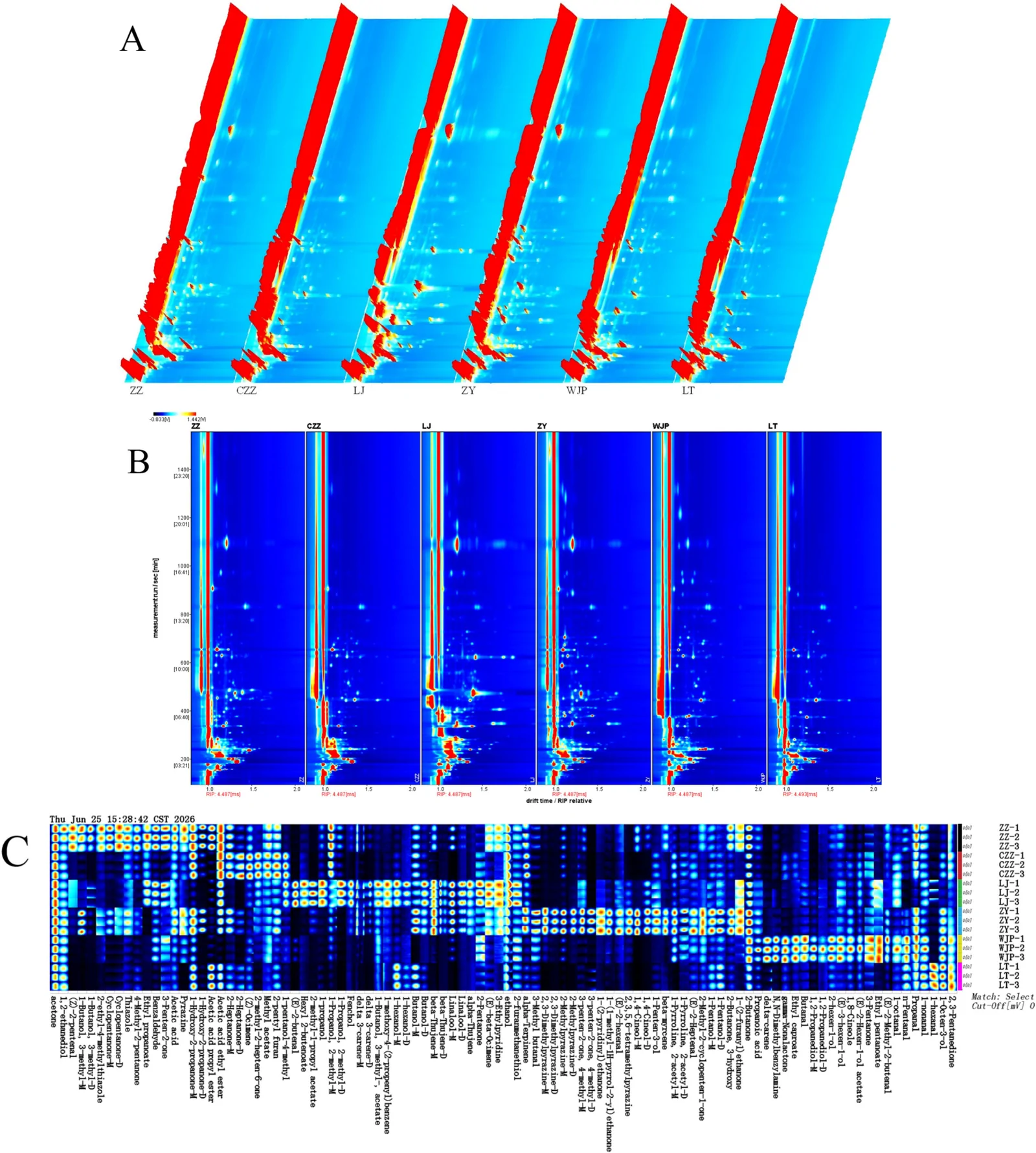
Gas chromatography-ion mobility spectrometry (GC-IMS) spectral characterization of braised chicken feet samples. (A) Three-dimensional topographic plots; (B) two-dimensional contour plots; (C) gallery fingerprint plot of volatile compounds.

**Table 3:** Relative contents of volatile compound categories across six braised chicken feet samples.

Category	ZZ	CZZ	LJ	ZY	WJP	LT
Aldehydes	6.31 ± 0.10^c^	5.26 ± 0.14^d^	7.15 ± 0.26^c^	4.93 ± 0.06^d^	14.10 ± 0.77^a^	9.19 ± 0.11^b^
Ketones	27.95 ± 0.47^b^	33.72 ± 0.46^a^	20.89 ± 0.16^c^	27.68 ± 0.25^b^	32.74 ± 0.95^a^	33.88 ± 0.24^a^
Alcohols	20.71 ± 0.72^c^	27.77 ± 0.21^a^	23.97 ± 0.27^b^	16.39 ± 0.08^d^	24.08 ± 0.78^b^	23.08 ± 0.41^b^
Esters	18.68 ± 0.32^b^	17.84 ± 0.22^c^	8.37 ± 0.12^d^	5.99 ± 0.29^e^	4.33 ± 0.22^f^	20.63 ± 0.18^a^
Pyrazines	2.13 ± 0.06^c^	2.12 ± 0.04^c^	1.01 ± 0.05^d^	7.97 ± 0.16^a^	2.68 ± 0.09^b^	0.75 ± 0.02^e^
Terpenes	18.55 ± 0.10^c^	7.75 ± 0.27^e^	34.15 ± 0.82^a^	29.75 ± 0.36^b^	14.51 ± 0.49^d^	7.95 ± 0.28^e^
Heterocyclic compounds	1.69 ± 0.02^d^	1.99 ± 0.05^c^	1.13 ± 0.02^e^	3.70 ± 0.08^a^	2.29 ± 0.03^b^	1.07 ± 0.03^e^
Sulfur-containing compounds	0.24 ± 0.00^c^	0.25 ± 0.02^c^	0.26 ± 0.01^c^	0.15 ± 0.01^d^	0.40 ± 0.01^a^	0.32 ± 0.02^b^
Acids	3.49 ± 0.15^a^	2.97 ± 0.05^abc^	2.29 ± 0.04^c^	3.10 ± 0.05^ab^	3.47 ± 0.59^a^	2.65 ± 0.10^bc^
Other compounds	0.26 ± 0.02^d^	0.33 ± 0.02^d^	0.78 ± 0.04^b^	0.33 ± 0.02^d^	1.40 ± 0.03^a^	0.49 ± 0.04^c^

Sample codes: ZZ, laboratory-prepared sample; CZZ, Caizhizhai; LJ, Liaoji Bangbangji; ZY, Ziyan Baiweiji; WJP, Wangjiapo; LT, Lintai. Data are expressed as mean ± SD (*n* = 3) of relative peak area percentages. Different superscript lowercase letters within the same row indicate significant differences among samples according to Tukey's HSD test (*P* < 0.05).

The gallery fingerprint (Fig. 3C) provided a compound-specific comparison of signal intensities across the six batches. Each row corresponds to one sample batch, and each column represents an individual volatile compound. The color scale reflects relative abundance, with red and orange blocks indicating higher relative abundance and dark blue indicating lower signal intensity or trace-level compounds. The horizontal color band patterns were highly reproducible within each sample, indicating satisfactory analytical repeatability among technical replicates. Clear differences in volatile composition were observed among samples. The laboratory-prepared sample exhibited relatively higher abundances of low-molecular-weight carbonyl compounds, whereas the commercial samples showed stronger signals for terpenes and pyrazine-type nitrogen heterocycles. The qualitative patterns observed in the fingerprint analysis were further supported by quantitative category-level analysis and multivariate modeling presented in Section 3.5.1.

#### 3.3.2 Category-specific volatile compound distribution

When the 81 identified volatile compounds were grouped by chemical class, ketones, alcohols, and terpenes were the three predominant categories (Fu et al., 2022; Liu et al., 2023), collectively accounting for 64.91% (LT) and 79.01% (LJ) of the total volatile profile (Table 3). Aldehydes and esters represented intermediate proportions, whereas each of the remaining five categories contributed less than 8% of the total volatile profile, with sulfur-containing compounds and other compounds each accounting for less than 1.5%. The predominance of ketones and alcohols may reflect their formation primarily through lipid oxidation, although their direct introduction from seasoning ingredients cannot be excluded.

Among the volatile compound classes, terpenes, pyrazines, and heterocyclic compounds exhibited the greatest variation among the six samples, making them important discriminating markers (Cheng et al., 2022; Sun et al., 2023). The relative abundance of terpenes varied over fourfold, ranging from 7.75% in CZZ to 34.15% in LJ. Pyrazines also showed substantial variation (ranging from 0.75% in LT to 7.97% in ZY), as did heterocyclic compounds (ranging from 1.07% in LT to 3.70% in ZY). Because these compound classes originate from distinctly different chemical pathways, terpenes from added spices, pyrazines primarily from the Maillard reaction, and heterocyclic compounds from both Maillard reactions and thermal degradation of sulfur-containing precursors, the uneven distribution of these classes among the six samples can be attributed to the relative contributions of these different pathways. In comparison, esters exhibited higher relative abundances in LT (20.63%), ZZ (18.68%), and CZZ (17.84%), which may be associated with the use of ethanol-containing marinade components (Yu et al., 2020). Alcohols showed relatively moderate variation among samples (16.39%–27.77%), whereas aldehydes exhibited greater variability, although they are commonly associated with lipid oxidation pathways. In particular, WJP exhibited the highest aldehyde content (14.10%), approximately threefold higher than that of ZY (4.93%). Overall, these category-level differences suggest that Maillard-derived heterocycles and spice-derived terpenes (Fu et al., 2022; Sun et al., 2023) are likely the major contributors to flavor differences among the samples.

### 3.4 Candidate aroma-active compound screening by ROAV

ROAV analysis identified 12 candidate aroma-active compounds (Liang et al., 2023; Nie et al., 2024; Yuan et al., 2024; Zhang, Dai, et al., 2025) (ROAV ≥1), including aldehydes (4), heterocyclic compounds (3), terpenes (2), alcohols (1), sulfur-containing compounds (1), and esters (1). A complete list of these compounds, together with their relative contents and ROAV values, is presented in Table 4. The number of detectable candidate aroma-active compounds varied among the six samples, ranging from five in LJ to ten in ZY.

**Table 4:** Relative contents and relative odor activity value (ROAV) values of volatile compounds identified in six braised chicken feet samples.

Compound	Relative content (Mean ± SD, %)						ROAV					
	ZZ	CZZ	LJ	ZY	WJP	LT	ZZ	CZZ	LJ	ZY	WJP	LT
3-Methylbutanal	0.11 ± 0.00^d^	0.07 ± 0.01^e^	0.02 ± 0.00^f^	0.32 ± 0.01^b^	0.37 ± 0.02^a^	0.17 ± 0.01^c^	2.21	1.41	0.41	10.79	4.61	2.68
Nonanal	0.32 ± 0.03^c^	0.33 ± 0.01^c^	0.23 ± 0.01^d^	0.17 ± 0.01^d^	0.50 ± 0.04^b^	0.57 ± 0.02^a^	1.35	1.34	0.87	1.14	1.24	1.78
(Z)-2-Pentenal	0.43 ± 0.01^a^	0.03 ± 0.00^d^	0.08 ± 0.01^b^	0.06 ± 0.00^c^	0.03 ± 0.01^de^	0.02 ± 0.00^f^	1.21	0.09	0.20	0.28	0.04	0.04
Propanal	2.12 ± 0.05^c^	2.31 ± 0.16^c^	0.46 ± 0.01^e^	1.71 ± 0.00^d^	3.38 ± 0.13^a^	2.83 ± 0.04^b^	0.95	0.99	0.19	1.21	0.90	0.93
Pentanal	1.06 ± 0.05^c^	0.80 ± 0.02^c^	0.19 ± 0.00^d^	0.95 ± 0.01^c^	4.61 ± 0.25^a^	3.54 ± 0.04^b^	0.37	0.27	0.06	0.53	0.96	0.92
Octanal	0.04 ± 0.00^b^	0.13 ± 0.02^a^	0.07 ± 0.01^b^	0.04 ± 0.00^b^	0.15 ± 0.03^a^	0.08 ± 0.00^b^	0.27	0.75	0.37	0.35	0.55	0.34
(E)-2-Butenal	0.23 ± 0.01^c^	0.24 ± 0.02^c^	4.48 ± 0.31^a^	0.18 ± 0.01^c^	1.14 ± 0.09^b^	0.25 ± 0.03^c^	0.04	0.04	0.74	0.05	0.12	0.03
Butanal	0.29 ± 0.01^c^	0.37 ± 0.06^bc^	0.38 ± 0.00^bc^	0.29 ± 0.00^c^	1.59 ± 0.11^a^	0.50 ± 0.01^b^	0.13	0.16	0.16	0.22	0.44	0.17
Hexanal	0.02 ± 0.00^c^	0.04 ± 0.01^c^	0.11 ± 0.01^b^	0.02 ± 0.00^c^	0.03 ± 0.00^c^	0.52 ± 0.02^a^	0.02	0.03	0.09	0.04	0.02	0.36
(E)-2-Hexenal	0.08 ± 0.00^b^	0.08 ± 0.00^b^	0.06 ± 0.01^b^	0.21 ± 0.03^a^	0.09 ± 0.01^b^	0.07 ± 0.00^b^	0.02	0.02	0.01	0.08	0.01	0.01
(E)-2-Heptenal	0.05 ± 0.00^bc^	0.11 ± 0.02^a^	0.05 ± 0.00^c^	0.14 ± 0.02^a^	0.10 ± 0.02^ab^	0.10 ± 0.02^a^	0.02	0.03	0.01	0.07	0.02	0.02
(E)-2-Methyl-2-butenal	0.47 ± 0.02^b^	0.46 ± 0.02^b^	0.14 ± 0.01^c^	0.39 ± 0.01^b^	1.09 ± 0.20^a^	0.29 ± 0.02^bc^	0.04	0.04	0.01	0.05	0.05	0.02
Benzaldehyde	1.08 ± 0.01^a^	0.29 ± 0.01^d^	0.89 ± 0.05^b^	0.44 ± 0.02^c^	1.03 ± 0.08^a^	0.25 ± 0.01^d^	0.01	–	0.01	0.01	0.01	–
3-Penten-2-one	0.24 ± 0.02^a^	0.06 ± 0.01^c^	0.16 ± 0.02^ab^	0.06 ± 0.01^c^	0.18 ± 0.07^ab^	0.13 ± 0.01^bc^	0.67	0.16	0.40	0.26	0.30	0.27
4-Methyl-3-penten-2-one	0.75 ± 0.01^c^	0.58 ± 0.02^d^	1.29 ± 0.08^b^	3.05 ± 0.07^a^	0.59 ± 0.05^d^	0.88 ± 0.02^c^	0.06	0.05	0.10	0.41	0.03	0.05
2-Heptanone	0.88 ± 0.02^bc^	2.19 ± 0.30^a^	0.13 ± 0.00^d^	0.83 ± 0.02^c^	0.27 ± 0.02^d^	1.25 ± 0.18^b^	0.03	0.06	–	0.04	–	0.03
2-Methyl-2-hepten-6-one	0.06 ± 0.00^e^	0.39 ± 0.01^a^	0.16 ± 0.01^b^	0.12 ± 0.01^c^	0.09 ± 0.01^d^	0.06 ± 0.00^e^	–	0.03	0.01	0.02	–	–
2,3-Pentanedione	0.08 ± 0.01^c^	0.12 ± 0.00^b^	0.04 ± 0.00^c^	0.07 ± 0.01^c^	0.16 ± 0.02^b^	0.22 ± 0.02^a^	0.01	0.02	0.01	0.02	0.01	0.02
Cyclopentanone	1.35 ± 0.02^a^	0.30 ± 0.03^c^	0.08 ± 0.01^f^	0.61 ± 0.02^b^	0.16 ± 0.01^e^	0.25 ± 0.01^d^	0.01	–	–	0.01	–	–
Acetone	12.48 ± 0.11^c^	15.87 ± 0.06^b^	12.10 ± 0.25^c^	10.76 ± 0.09^d^	16.41 ± 0.45^ab^	16.93 ± 0.10^a^	–	–	–	–	–	–
4-Methyl-2-pentanone	0.21 ± 0.02^a^	0.09 ± 0.00^b^	0.03 ± 0.00^d^	0.06 ± 0.00^c^	0.04 ± 0.00^cd^	0.11 ± 0.00^b^	–	–	–	–	–	–
1-Hydroxy-2-propanone	5.79 ± 0.30^a^	6.08 ± 0.29^a^	0.55 ± 0.01^d^	4.28 ± 0.05^b^	2.44 ± 0.12^c^	5.50 ± 0.28^a^	–	–	–	–	–	–
2-Pentanone	0.14 ± 0.02^f^	0.94 ± 0.04^c^	1.15 ± 0.03^b^	0.54 ± 0.03^d^	1.60 ± 0.07^a^	0.42 ± 0.02^e^	–	–	–	–	–	–
2-Methyl-2-cyclopenten-1-one	0.05 ± 0.00^d^	0.03 ± 0.00^e^	0.02 ± 0.00^e^	0.09 ± 0.00^b^	0.10 ± 0.01^a^	0.07 ± 0.00^c^	–	–	–	–	–	–
3-Hydroxy-2-butanone	0.51 ± 0.03^a^	0.25 ± 0.00^c^	0.09 ± 0.01^d^	0.52 ± 0.02^a^	0.26 ± 0.01^c^	0.32 ± 0.00^b^	–	–	–	–	–	–
2-Butanone	5.30 ± 0.13^d^	6.43 ± 0.08^c^	4.85 ± 0.04^d^	6.49 ± 0.04^c^	9.74 ± 0.40^a^	7.55 ± 0.08^b^	–	–	–	–	–	–
3-Pentanone	0.11 ± 0.01^e^	0.38 ± 0.00^b^	0.24 ± 0.01^c^	0.21 ± 0.01^cd^	0.70 ± 0.05^a^	0.18 ± 0.00^d^	–	–	–	–	–	–
1-Octen-3-ol	0.10 ± 0.00^e^	0.35 ± 0.01^d^	0.12 ± 0.01^e^	0.38 ± 0.01^c^	0.66 ± 0.01^b^	0.80 ± 0.01^a^	0.43	1.43	0.45	2.52	1.64	2.49
3-Methyl-1-butanol	2.09 ± 0.44^a^	0.29 ± 0.02^c^	0.56 ± 0.02^bc^	0.92 ± 0.03^b^	0.15 ± 0.02^c^	0.19 ± 0.02^c^	0.04	–	0.01	0.02	–	–
1-Butanol	0.60 ± 0.03^d^	0.94 ± 0.06^b^	0.73 ± 0.03^c^	0.94 ± 0.04^b^	0.09 ± 0.00^e^	1.29 ± 0.04^a^	–	0.01	0.01	0.01	–	0.01
2-Hexen-1-ol	0.10 ± 0.01^c^	0.09 ± 0.01^c^	0.06 ± 0.01^d^	0.06 ± 0.01^d^	0.35 ± 0.00^a^	0.18 ± 0.00^b^	–	–	–	–	0.01	0.01
(*E*)-3-Hexen-1-ol	0.05 ± 0.01^b^	0.06 ± 0.01^b^	0.06 ± 0.01^b^	0.04 ± 0.01^b^	0.17 ± 0.01^a^	0.06 ± 0.01^b^	–	–	–	–	0.01	–
Ethane-1,2-diol	0.97 ± 0.16^c^	1.08 ± 0.08^c^	0.67 ± 0.04^d^	0.88 ± 0.04^cd^	1.31 ± 0.03^b^	1.66 ± 0.04^a^	–	–	–	–	–	–
4-Methyl-1-pentanol	0.05 ± 0.00^d^	0.39 ± 0.02^a^	0.21 ± 0.01^b^	0.07 ± 0.00^d^	0.12 ± 0.02^c^	0.08 ± 0.01^d^	–	–	–	–	–	–
1-Propanol	0.39 ± 0.02^b^	0.36 ± 0.02^b^	1.64 ± 0.00^a^	0.13 ± 0.01^d^	0.15 ± 0.01^d^	0.24 ± 0.00^b^	–	–	–	–	–	–
2-Methyl-1-Propanol	3.07 ± 0.03^b^	3.09 ± 0.10^b^	3.34 ± 0.02^a^	0.89 ± 0.01^d^	1.18 ± 0.07^c^	0.41 ± 0.01^e^	–	–	–	–	–	–
1-Hexanol	0.08 ± 0.01^d^	0.17 ± 0.01^c^	0.41 ± 0.03^b^	0.09 ± 0.00^d^	0.17 ± 0.01^c^	0.66 ± 0.01^a^	–	–	–	–	–	–
Ethanol	12.49 ± 0.17^c^	19.99 ± 0.32^a^	14.85 ± 0.04^b^	10.50 ± 0.01^d^	12.90 ± 0.67^c^	15.63 ± 0.11^b^	–	–	–	–	–	–
1-Penten-3-ol	0.04 ± 0.00^d^	0.12 ± 0.00^b^	0.10 ± 0.01^b^	0.20 ± 0.02^a^	0.02 ± 0.00^d^	0.07 ± 0.01^c^	–	–	–	–	–	–
1-Pentanol	0.26 ± 0.02^d^	0.34 ± 0.01^c^	0.22 ± 0.02^e^	0.52 ± 0.02^b^	0.29 ± 0.02^d^	0.60 ± 0.01^a^	–	–	–	–	–	–
Propane-1,2-diol	0.41 ± 0.02^b^	0.49 ± 0.04^b^	1.02 ± 0.24^b^	0.78 ± 0.04^b^	6.51 ± 1.60^a^	1.21 ± 0.20^b^	–	–	–	–	–	–
Ethyl hexanoate	0.04 ± 0.01^d^	0.14 ± 0.01^b^	0.06 ± 0.00^c^	0.05 ± 0.01^cd^	0.47 ± 0.00^a^	0.12 ± 0.01^b^	0.17	0.56	0.24	0.33	1.17	0.38
3-Methylbutyl acetate	0.03 ± 0.00^d^	0.05 ± 0.00^d^	0.25 ± 0.01^a^	0.12 ± 0.01^c^	0.23 ± 0.04^ab^	0.18 ± 0.01^b^	0.07	0.10	0.48	0.41	0.28	0.28
(E)-Hexen-1-ol acetate	0.13 ± 0.02^c^	0.51 ± 0.07^b^	0.38 ± 0.02^bc^	0.21 ± 0.01^bc^	1.04 ± 0.25^a^	0.49 ± 0.08^b^	0.05	0.21	0.14	0.14	0.26	0.15
γ-Nonalactone	0.03 ± 0.00^c^	0.17 ± 0.01^b^	0.04 ± 0.00^c^	0.03 ± 0.00^c^	0.57 ± 0.02^a^	0.19 ± 0.01^b^	0.02	0.11	0.02	0.03	0.22	0.09
Ethyl propanoate	0.22 ± 0.02^a^	0.17 ± 0.01^b^	0.18 ± 0.00^b^	0.01 ± 0.00^c^	0.03 ± 0.00^c^	0.02 ± 0.00^c^	0.09	0.07	0.07	0.01	0.01	0.01
Ethyl pentanoate	0.01 ± 0.00^d^	0.01 ± 0.00^cd^	0.02 ± 0.00^b^	0.01 ± 0.00^c^	0.08 ± 0.00^a^	0.01 ± 0.00^cd^	0.01	0.01	0.01	0.02	0.04	0.01
Propyl acetate	6.25 ± 0.27^b^	0.72 ± 0.04^cd^	0.67 ± 0.02^d^	0.78 ± 0.02^cd^	1.02 ± 0.04^c^	10.30 ± 0.08^a^	0.01	–	–	–	–	0.02
2-Methylpropyl acetate	0.01 ± 0.00^c^	0.02 ± 0.00^c^	0.29 ± 0.02^a^	0.07 ± 0.01^b^	0.03 ± 0.00^c^	0.03 ± 0.00^c^	–	–	0.02	0.01	–	–
Ethyl acetate	11.80 ± 0.07^b^	15.79 ± 0.10^a^	6.19 ± 0.13^d^	4.61 ± 0.25^e^	0.73 ± 0.05^f^	9.19 ± 0.16^c^	0.01	0.01	–	0.01	–	0.01
Methyl acetate	0.12 ± 0.01^b^	0.20 ± 0.01^a^	0.08 ± 0.01^cd^	0.07 ± 0.00^d^	0.10 ± 0.01^c^	0.05 ± 0.01^e^	–	–	–	–	–	–
Hexyl 2-butenoate	0.03 ± 0.00^cd^	0.06 ± 0.01^b^	0.21 ± 0.01^a^	0.02 ± 0.00^d^	0.05 ± 0.01^bc^	0.05 ± 0.01^bc^	–	–	–	–	–	–
2,3-Dimethylpyrazine	0.16 ± 0.00^cd^	0.18 ± 0.01^c^	0.10 ± 0.01^d^	2.23 ± 0.05^a^	0.58 ± 0.03^b^	0.12 ± 0.00^cd^	–	–	–	0.01	–	–
Pyrazine	0.36 ± 0.06^a^	0.21 ± 0.01^b^	0.08 ± 0.00^c^	0.32 ± 0.02^a^	0.22 ± 0.01^b^	0.02 ± 0.00^c^	–	–	–	–	–	–
2-Methylpyrazine	1.52 ± 0.02^b^	1.62 ± 0.03^b^	0.43 ± 0.01^c^	4.36 ± 0.07^a^	1.54 ± 0.06^b^	0.41 ± 0.02^c^	–	–	–	–	–	–
2,3,5,6-Tetramethylpyrazine	0.09 ± 0.01^d^	0.11 ± 0.01^cd^	0.40 ± 0.05^b^	1.06 ± 0.07^a^	0.33 ± 0.03^b^	0.20 ± 0.01^c^	–	–	–	–	–	–
β-Thujene	2.39 ± 0.13^c^	1.06 ± 0.02^d^	8.00 ± 0.18^a^	6.19 ± 0.10^b^	1.08 ± 0.12^d^	0.82 ± 0.05^d^	5.01	2.13	15.27	20.63	1.35	1.28
Linalool	9.66 ± 0.16^c^	2.22 ± 0.03^f^	19.07 ± 0.13^a^	14.59 ± 0.24^b^	6.61 ± 0.20^d^	3.03 ± 0.09^e^	6.75	1.49	12.12	16.18	2.75	1.57
Fenchol	0.67 ± 0.02^b^	0.30 ± 0.02^d^	2.13 ± 0.05^a^	0.74 ± 0.03^b^	0.47 ± 0.01^c^	0.70 ± 0.06^b^	0.18	0.07	0.51	0.31	0.07	0.14
1,4-Cineole	2.27 ± 0.31^b^	0.24 ± 0.02^d^	0.66 ± 0.01^c^	4.45 ± 0.05^a^	0.23 ± 0.01^d^	0.20 ± 0.01^d^	0.08	0.01	0.02	0.25	–	0.01
1,8-Cineole	0.11 ± 0.01^d^	0.40 ± 0.06^b^	0.17 ± 0.00^cd^	0.38 ± 0.03^bc^	1.08 ± 0.17^a^	0.47 ± 0.07^b^	0.04	0.13	0.05	0.21	0.23	0.12
β-Myrcene	0.34 ± 0.04^ab^	0.22 ± 0.06^cd^	0.08 ± 0.01^e^	0.45 ± 0.08^a^	0.12 ± 0.00^de^	0.24 ± 0.02^bc^	0.10	0.06	0.02	0.20	0.02	0.05
(*Z*)-β-Ocimene	0.45 ± 0.03^c^	1.22 ± 0.02^a^	0.23 ± 0.02^d^	0.16 ± 0.00^e^	0.47 ± 0.02^c^	0.57 ± 0.03^b^	0.06	0.14	0.03	0.03	0.03	0.05
α-Thujene	0.04 ± 0.00^c^	0.05 ± 0.01^c^	0.15 ± 0.03^a^	0.10 ± 0.00^b^	0.05 ± 0.00^c^	0.06 ± 0.01^c^	0.03	0.04	0.12	0.13	0.03	0.04
(*E*)-β-Ocimene	0.19 ± 0.00^b^	0.09 ± 0.01^c^	0.27 ± 0.01^a^	0.19 ± 0.03^b^	0.09 ± 0.01^c^	0.13 ± 0.01^c^	0.02	0.01	0.03	0.04	0.01	0.01
α-Terpinene	0.56 ± 0.06^c^	1.00 ± 0.06^a^	0.28 ± 0.01^e^	0.76 ± 0.03^b^	0.27 ± 0.01^e^	0.44 ± 0.00^d^	–	–	–	0.01	–	–
δ-3-Carene	1.79 ± 0.05^b^	0.76 ± 0.03^cd^	2.86 ± 0.81^a^	1.58 ± 0.08^bc^	0.59 ± 0.01^d^	0.67 ± 0.01^d^	0.01	–	0.01	0.01	–	–
δ-Carene	0.07 ± 0.01^c^	0.18 ± 0.04^c^	0.25 ± 0.05^c^	0.18 ± 0.01^c^	3.46 ± 0.20^a^	0.62 ± 0.05^b^	–	–	–	–	0.01	–
2-Acetyl-1-pyrroline	0.34 ± 0.01^c^	1.00 ± 0.04^b^	0.34 ± 0.02^c^	1.07 ± 0.03^b^	1.19 ± 0.07^a^	0.17 ± 0.01^d^	14.30	40.16	13.10	71.53	29.59	5.29
2-Ethyl-4-methylthiazole	0.13 ± 0.01^a^	0.04 ± 0.00^c^	0.01 ± 0.00^d^	0.07 ± 0.00^b^	0.04 ± 0.00^c^	0.02 ± 0.00^d^	10.06	2.68	1.03	8.00	1.76	1.30
1-(1-Methyl-1H-pyrrol-2-yl)ethanone	0.28 ± 0.02^c^	0.30 ± 0.02^c^	0.31 ± 0.05^c^	1.70 ± 0.03^a^	0.60 ± 0.08^b^	0.34 ± 0.02^c^	0.12	0.12	0.12	1.13	0.15	0.10
2-Pentylfuran	0.10 ± 0.01^bc^	0.19 ± 0.01^a^	0.06 ± 0.01^d^	0.09 ± 0.01^c^	0.06 ± 0.01^d^	0.12 ± 0.00^b^	0.07	0.13	0.04	0.10	0.02	0.06
3-Ethylpyridine	0.06 ± 0.01^a^	0.03 ± 0.00^d^	0.06 ± 0.00^a^	0.05 ± 0.00^b^	0.03 ± 0.00^cd^	0.04 ± 0.00^bc^	0.11	0.04	0.10	0.13	0.03	0.05
Thiazole	0.61 ± 0.04^a^	0.36 ± 0.01^b^	0.22 ± 0.00^c^	0.34 ± 0.03^b^	0.26 ± 0.01^c^	0.26 ± 0.01^c^	0.04	0.02	0.01	0.03	0.01	0.01
1-(2-Pyridinyl)ethanone	0.05 ± 0.00^cd^	0.05 ± 0.00^cd^	0.03 ± 0.00^d^	0.28 ± 0.02^a^	0.07 ± 0.00^bc^	0.08 ± 0.01^b^	–	–	–	0.03	–	–
1-(2-Furanyl)ethanone	0.12 ± 0.01^a^	0.03 ± 0.00^e^	0.09 ± 0.00^b^	0.10 ± 0.01^b^	0.05 ± 0.01^c^	0.04 ± 0.00^de^	–	–	–	–	–	–
2-Furanmethanethiol	0.24 ± 0.00^c^	0.25 ± 0.02^c^	0.26 ± 0.01^c^	0.15 ± 0.01^d^	0.40 ± 0.01^a^	0.32 ± 0.02^b^	100.00	100.00	100.00	100.00	100.00	100.00
Acetic acid	2.69 ± 0.16^a^	2.49 ± 0.04^a^	1.73 ± 0.02^c^	2.11 ± 0.02^b^	1.78 ± 0.21^c^	1.81 ± 0.05^bc^	–	–	–	–	–	–
Propanoic acid	0.80 ± 0.01^bc^	0.48 ± 0.01^c^	0.56 ± 0.02^bc^	0.99 ± 0.03^b^	1.69 ± 0.39^a^	0.84 ± 0.07^bc^	–	–	–	–	–	–
1-Methoxy-4-(2-propenyl)benzene	0.13 ± 0.01^d^	0.19 ± 0.00^c^	0.67 ± 0.02^a^	0.22 ± 0.01^c^	0.40 ± 0.03^b^	0.36 ± 0.04^b^	0.05	0.08	0.26	0.14	0.10	0.11
*N*,*N*-Dimethylbenzylamine	0.13 ± 0.02^b^	0.14 ± 0.02^b^	0.11 ± 0.06^b^	0.11 ± 0.02^b^	0.99 ± 0.03^a^	0.13 ± 0.01^b^	0.01	0.01	0.01	0.02	0.05	0.01

Different superscript lowercase letters within the same row indicate significant differences among samples according to Tukey's HSD test (*P* < 0.05). 2-Furanmethanethiol was used as the reference compound in the ROAV calculation; its ROAV was consequently normalized to 100 in all six samples, which does not imply equal concentration or an absolute aroma contribution.

Heterocyclic compounds and terpenes were the major contributors to aroma differences among the six samples. 2-Acetyl-1-pyrroline, which imparts a popcorn-like aroma (Cai et al., 2024; Dach & Schieberle, 2021), exhibited substantial variation in relative content, ranging from the lowest level in LT to the highest level in WJP, and achieved the highest ROAV among the candidate compounds apart from the reference 2-furanmethanethiol (ROAV = 100). 2-Ethyl-4-methylthiazole, characterized by roasted, nutty aroma notes, reached its highest ROAV values in ZZ and ZY, but exhibited relatively low ROAV values in LJ and LT (1.03 and 1.30, respectively). 1-(1-Methyl-1H-pyrrol-2-yl)ethanone, an earthy, nitrogen-containing compound, was detected in all samples and served as an important discriminative marker. Among the terpenes, β-thujene and linalool (Sohail et al., 2022) exhibited the highest ROAV values in LJ and ZY, whereas their contributions were significantly lower in the other samples.

Strecker degradation products, lipid oxidation products, and esters also contributed to the overall aroma profile. 3-Methylbutanal, a Strecker degradation aldehyde characterized by malty and cocoa-like notes (Sohail et al., 2022; Yue et al., 2024), exhibited its highest ROAV in ZY (10.79) but showed relatively low values in the other samples. Nonanal, a characteristic fatty, citrus-like aldehyde (Han et al., 2023; Sohail et al., 2022), showed moderate ROAV values across all samples, indicating that lipid oxidation contributed consistently to the fatty aroma background. Among the short-chain aldehydes, propanal (ethereal, pungent) exhibited an ROAV ≥1 only in ZY, pentanal (green, woody) remained below an ROAV of 1 in all samples, and (Z)-2-pentenal (fruity, green) reached an ROAV ≥1 only in ZZ. 1-Octen-3-ol, a mushroom-like alcohol (Han et al., 2023; Yue et al., 2024) formed through arachidonic acid oxidation, exceeded the ROAV threshold in CZZ, ZY, WJP, and LT, suggesting differences in lipid oxidation characteristics among the samples. Ethyl hexanoate (fruity, wine-like) was the only ester with an ROAV ≥1, exceeding the threshold only in WJP (1.17).

One-way ANOVA revealed significant differences among the six samples for all 12 candidate aroma-active compounds (*P* < 0.05). Collectively, the ROAV results reveal that the characteristic aroma profile of braised chicken feet is determined not by a single dominant class of volatile compounds but by the relative contributions of heterocyclic Maillard reaction products, spice-derived terpenes, and lipid-derived aldehydes. Differences in the relative contributions of these three compound classes appear to be the primary factors distinguishing the aroma profiles of the different samples.

### 3.5 Multivariate discrimination and visualization

#### 3.5.1 PCA and OPLS-DA

To provide an overview of the volatile profile, PCA and OPLS-DA were first applied to all 81 detected volatiles (Fig. S1, S2). In both models, CZZ, LJ, LT, and WJP were clearly separated, whereas ZZ and ZY overlapped and could not be fully resolved (OPLS-DA: R^2^Y = 0.998, Q^2^ = 0.997; permutation R^2^ = 0.181, Q^2^ = −0.843). Unsupervised PCA was then performed using the 12 candidate aroma-active compounds (ROAV ≥1) (Fig. 4A). The first two principal components explained 69.6% of the total variance (R^2^X[1] = 0.468, R^2^X[2] = 0.228). The tight within-group clustering of the triplicate measurements indicated good analytical reproducibility, whereas the clear separation among sample groups along both PC1 and PC2 demonstrated distinct differences in their volatile profiles. The PCA score plot broadly separated the samples into three groupings: ZY, LJ, and ZZ on the left side of PC1, CZZ in an intermediate position, and WJP and LT on the right. The three samples on the left were further separated along PC2, with ZY positioned at the top of the plot, LJ near the horizontal axis, and ZZ at the bottom.

**Fig. 4:**
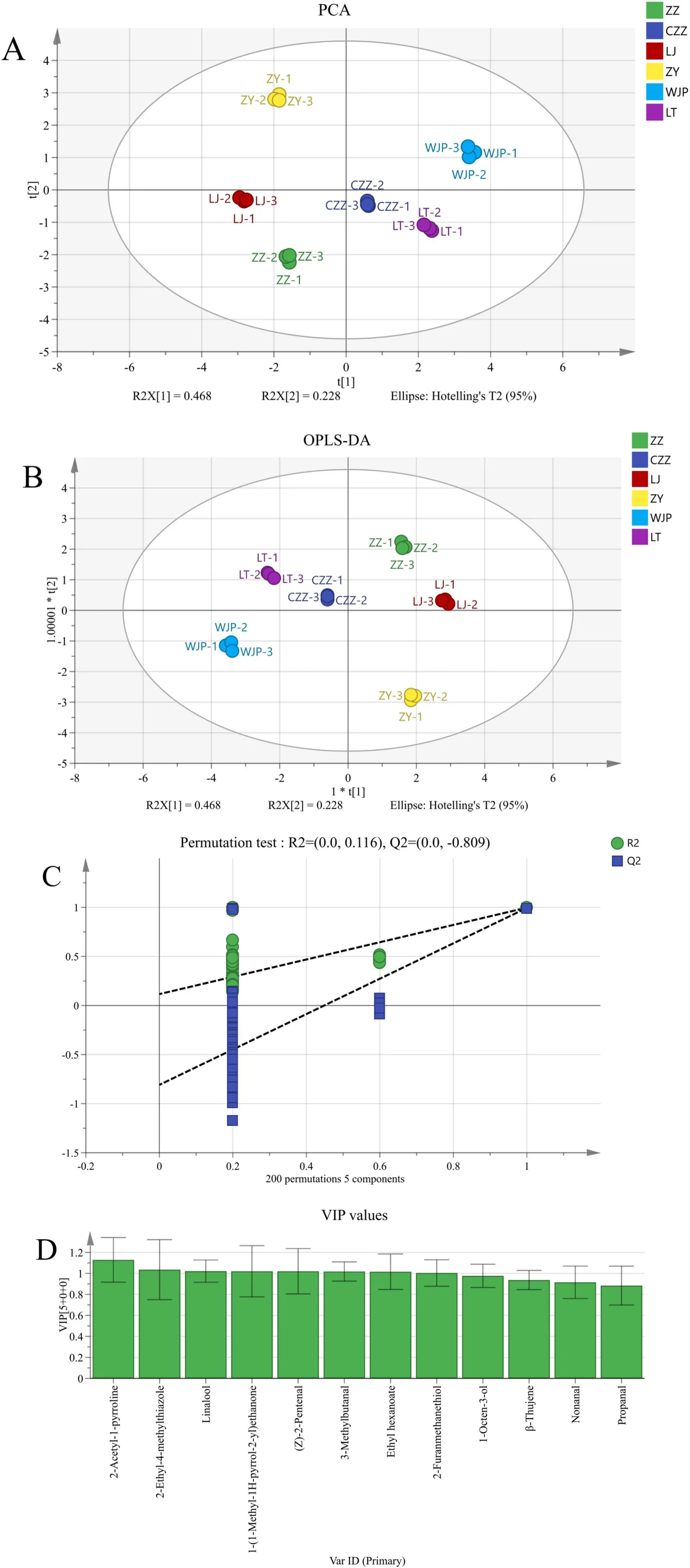
Multivariate statistical analysis of volatile compounds in six braised chicken feet samples. (A) Principal component analysis (PCA) score plot; (B) orthogonal partial least squares discriminant analysis (OPLS-DA) score plot; (C) permutation test; (D) variable importance in projection (VIP) bar chart.

Supervised OPLS-DA was then performed using the same 12 compounds to further enhance discrimination (Fig. 4B). The model completely separated all six samples, including ZZ and ZY, and exhibited strong explanatory and predictive power (R^2^Y = 0.996, Q^2^ = 0.991). Model validation using a 200-permutation test (Fig. 4C) yielded intercept values of R^2^ = 0.116 and Q^2^ = −0.809, indicating that the model was not overfitted. In contrast to the 81-compound model, the 12-compound model fully resolved ZZ and ZY. This difference likely reflects the composition of the two variable sets: 69 of the 81 compounds had ROAV below 1, forming a background largely shared among samples, whereas the 12 compounds with ROAV ≥1 showed greater variation between ZZ and ZY. Under unit-variance scaling, greater weight is assigned to the shared background compounds, which may attenuate the ZZ–ZY separation in the full model. Because the 12 candidate compounds were selected using ROAV—a threshold-based criterion derived from odor activity rather than from the multivariate models—the subsequent analyses focused on these candidate aroma-active compounds.

The VIP scores (Fig. 4*D*) ranked the contribution of each compound to the OPLS-DA model. Eight compounds exhibited VIP scores greater than 1.0 (Yu et al., 2025), the conventional threshold for variables with important discriminatory power, whereas the remaining four compounds, 1-octen-3-ol, β-thujene, nonanal, and propanal, showed VIP scores below 1.0, suggesting a comparatively limited contribution to discrimination among the samples. The three highest-ranked compounds were 2-acetyl-1-pyrroline, 2-ethyl-4-methylthiazole, and linalool, representing Maillard-derived heterocyclic compounds and spice-derived terpenes, consistent with the multiple pathways contributing to aroma differentiation described above. Notably, three of these four compounds (nonanal, propanal, and 1-octen-3-ol) are primarily associated with lipid oxidation, suggesting that they contribute to the common fatty aroma background shared by all samples rather than to discrimination among the samples, despite the low intramuscular fat content of chicken feet (Zhan et al., 2025). The relatively high VIP score of linalool further highlights the contribution of spice-derived terpenes, particularly in LJ and ZY, where this compound also exhibited its highest ROAV values.

#### 3.5.2 Distribution patterns of candidate aroma-active compounds

Hierarchical clustering based on the normalized ROAV values of the 12 candidate aroma-active compounds revealed distinct differentiation patterns among the six samples (Fig. 5). ZZ and LJ exhibited the highest similarity and formed the first close sub-cluster, whereas CZZ and WJP constituted a second sub-cluster. The CZZ–WJP sub-cluster was subsequently joined by LT, and this three-sample cluster then merged with the ZZ–LJ sub-cluster to form a five-sample cluster. In contrast, ZY exhibited the greatest distinctiveness, appearing as the most distant sample with the largest clustering distance from the other samples. The heatmap further showed that the two spice-related samples, LJ and ZY, did not cluster closely, indicating significant differences in their overall volatile profiles.

**Fig. 5:**
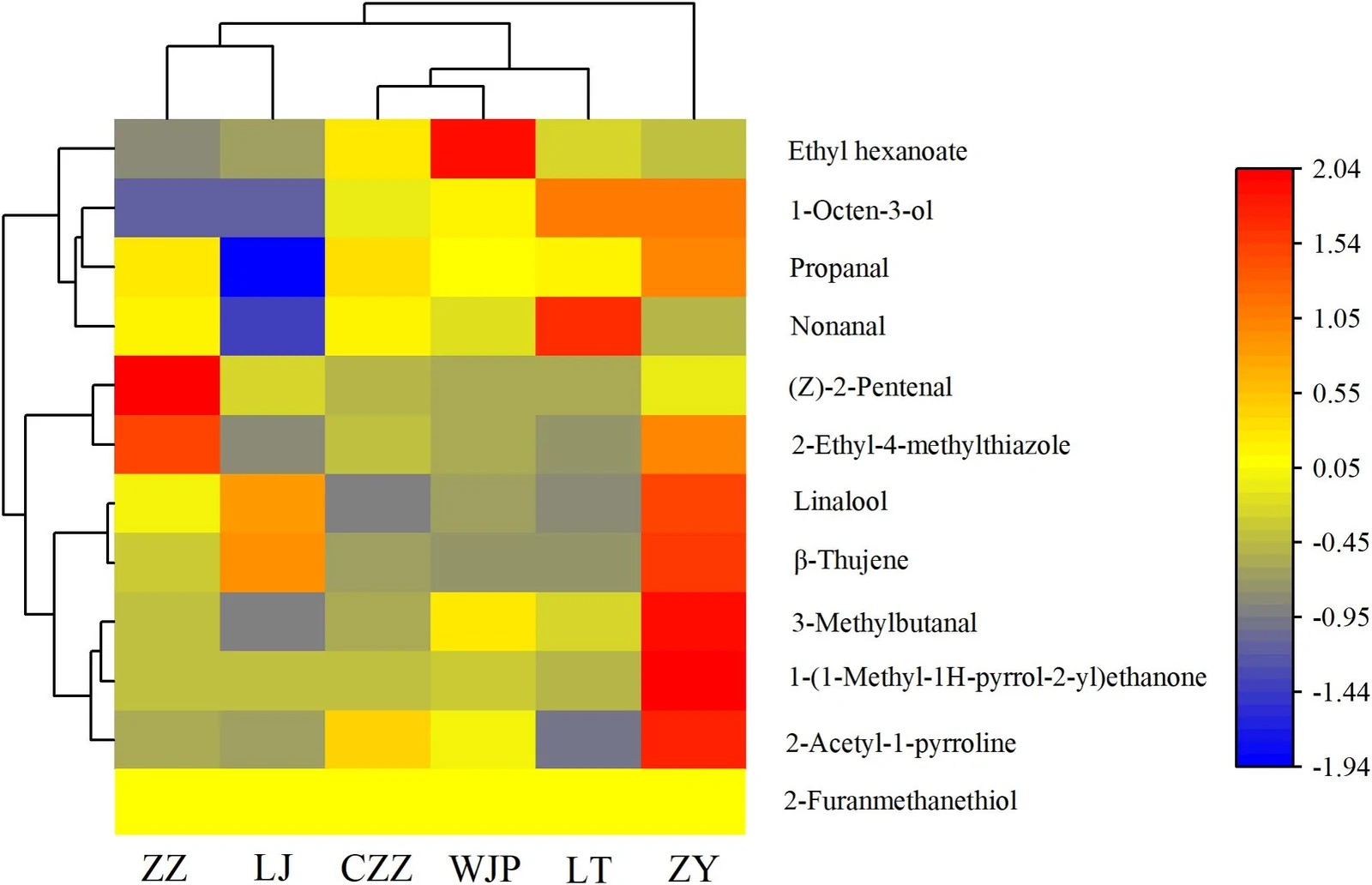
Heatmap clustering of candidate aroma-active compounds in six braised chicken feet samples.

Row-wise clustering grouped the 12 compounds into two functional modules corresponding to distinct aroma formation pathways. Module I comprised ethyl hexanoate, 1-octen-3-ol, propanal, and nonanal, which are primarily associated with lipid oxidation and esterification pathways; ethyl hexanoate and nonanal peaked in WJP and LT, respectively, whereas 1-octen-3-ol and propanal were most prominent in ZY. Module II included (Z)-2-pentenal, the terpenes β-thujene and linalool, the Strecker degradation aldehyde 3-methylbutanal, the sulfur-containing compound 2-furanmethanethiol, and the Maillard-derived heterocyclic compounds 2-ethyl-4-methylthiazole, 1-(1-methyl-1H-pyrrol-2-yl)ethanone, and 2-acetyl-1-pyrroline. Although (Z)-2-pentenal is also a lipid-derived aldehyde, its ROAV peaked in the laboratory-prepared ZZ sample, and its clustering pattern therefore resembled the spice- and Maillard-related compounds rather than the bulk lipid oxidation markers. The terpenes β-thujene and linalool showed their highest ROAVs in the LJ and ZY samples, and 1-(1-methyl-1H-pyrrol-2-yl)ethanone was most prominent in ZY. The Maillard-derived heterocycles exhibited a more dispersed distribution, with the ROAV of 2-acetyl-1-pyrroline peaking in ZY, that of 2-ethyl-4-methylthiazole in ZZ, and 2-furanmethanethiol (the reference compound) showing a constant value across all samples. ZZ exhibited intermediate ROAVs for most compounds from both modules. The clear separation between lipid-derived compounds and spice/Maillard-related compounds highlights a key feature of flavor formation in braised chicken feet: the characteristic aroma profiles are largely reflected by the relative contributions of endogenous lipid oxidation and exogenous spice-derived and Maillard reaction products, with sample differentiation arising from their combined effects rather than from individual compounds alone (Fu et al., 2022; Han et al., 2023).

#### 3.5.3 Flavor wheel construction

The flavor wheel (Fig. 6) provided a sensory-oriented visualization of the 81 volatile compounds by assigning them to aroma descriptors obtained from published odor databases (Sohail et al., 2022). The compounds were distributed among eleven primary aroma categories: Musty, Floral, Pungent, Green, Fruity, Herbal, Spicy, Fatty, Camphor, Almond, and Solvent. This classification facilitated the interpretation of the volatile composition in terms of the odor descriptors annotated in these databases and highlighted the major volatile compound groups underlying the overall aroma profile. It should be noted that these aroma descriptors were derived from published databases rather than from the directly evaluated sensory attributes of this study, which were limited to appearance, flavor, mouthfeel, and taste.

**Fig. 6:**
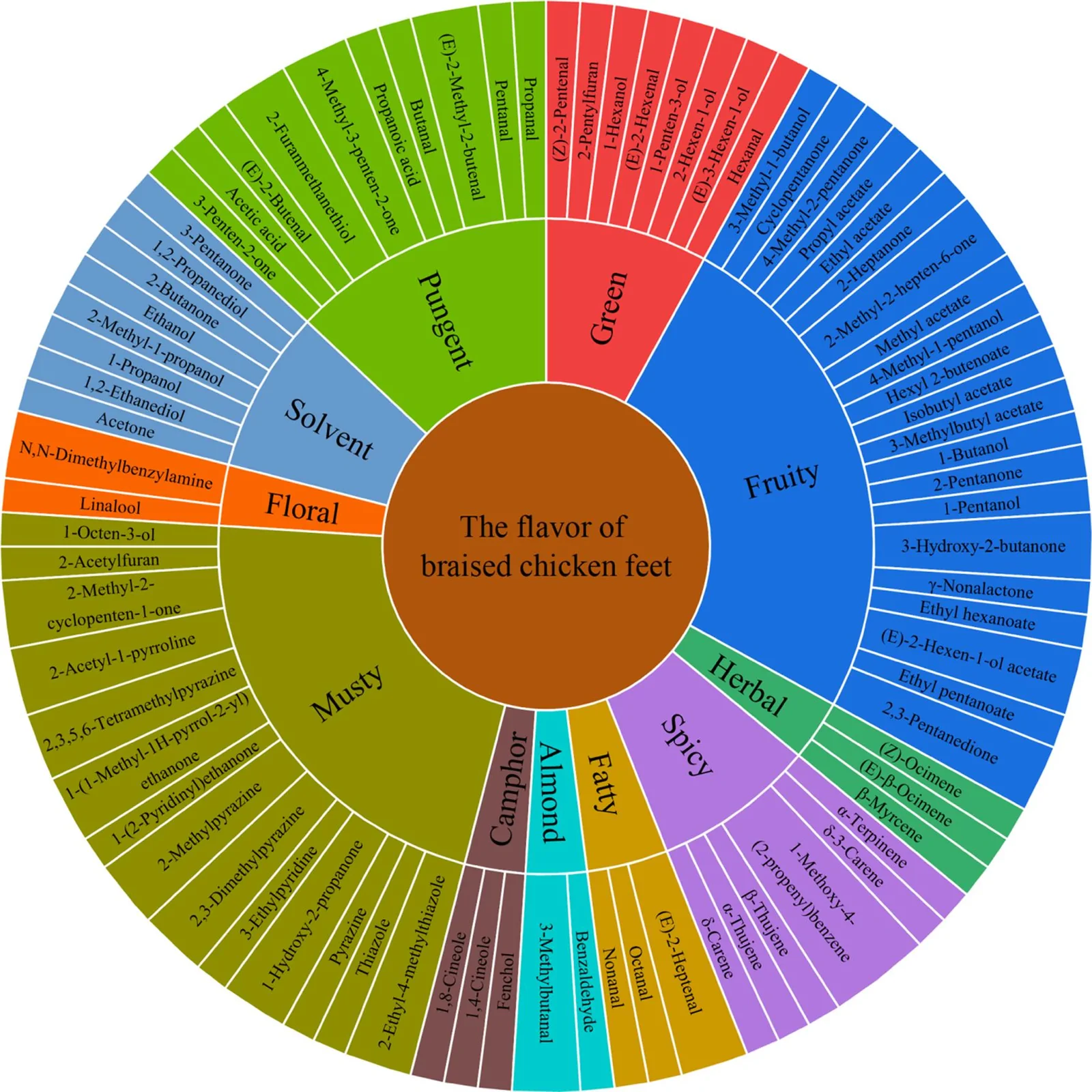
Flavor wheel of braised chicken feet samples.

The Fatty, Green, and Pungent categories were primarily associated with lipid-derived volatile compounds. Short-chain aldehydes, such as propanal and pentanal, were grouped into the Pungent category and contributed sharp, stimulating aroma notes. Medium- and long-chain aldehydes, particularly nonanal, represented major contributors to the Fatty category and were associated with fatty, citrus-like aroma characteristics across all samples at moderate ROAV levels. The Green category was mainly represented by C6 and C7 aldehydes and alcohols, including hexanal and (E)-2-hexenal, which contributed fresh, grassy, and leafy aroma notes. These lipid-derived compounds were detected in all six samples, suggesting that lipid oxidation provides a common chemical basis for the odor categories annotated to these compounds. However, their relative contributions varied considerably among the six samples. For example, 1-octen-3-ol, an earthy, mushroom-like compound categorized in the Musty category, exhibited the lowest ROAV in the laboratory-prepared ZZ sample and the highest ROAV in the ZY and LT samples, indicating that the contribution of this lipid-derived volatile to the overall aroma differed among the six samples.

The Herbal, Spicy, and Floral categories were primarily associated with spice-derived terpenoid compounds (Liu et al., 2023; Sun et al., 2023). Terpenes such as β-thujene, ocimenes, and myrcene contributed mainly to the Herbal and Spicy categories, while linalool was a major contributor to the Floral category. The relative abundances of β-thujene and linalool, the two predominant terpenes, varied substantially among the six samples and were the highest in the LJ and ZY samples. The Musty category was primarily represented by Maillard reaction-derived heterocyclic compounds (Yu, Zhang, et al., 2021), including 2-acetyl-1-pyrroline (popcorn-like aroma), 2-ethyl-4-methylthiazole (roasted, nutty aroma), and 1-(1-methyl-1H-pyrrol-2-yl)ethanone (earthy aroma), as well as several pyrazine derivatives. These heterocyclic compounds followed a strongly sample-dependent variation. For example, 2-acetyl-1-pyrroline showed the highest relative content in WJP, whereas 2-ethyl-4-methylthiazole reached its highest level in the laboratory-prepared ZZ sample. These findings indicate that the balance between spice-derived and Maillard reaction-derived volatile compounds is associated with the distinct odor profiles annotated for each sample. Overall, the flavor wheel transformed the complex volatile dataset into an integrated odor-based interpretation, highlighting that lipid-derived compounds contribute to a shared chemical foundation of the annotated odor profile, while variations in spice-derived and Maillard reaction-derived volatile compounds are associated with differences in sample-specific odor profiles.

### 3.6 Correlation and pathway integration

#### 3.6.1 Pearson correlation analysis

To investigate the relationships between the instrumental measurements and sensory attributes, Pearson correlation analysis was performed between 11 of the 12 candidate aroma-active compounds and the four sensory attributes (Fig. 7). 2-Furanmethanethiol was excluded from this analysis because its ROAV was normalized to a constant value of 100 in all six samples and therefore showed no variance. Among the 11 compounds, only two showed a nominally significant positive correlation with a single sensory attribute (*P* < 0.05): (Z)-2-pentenal with mouthfeel and nonanal with flavor (*r* = 0.84 and 0.83, respectively). Because these correlations were computed from six sample means (*n* = 6, *df* = 4), the significance threshold at *P* < 0.05 is *r* ≈ 0.81; the two nominally significant correlations were therefore marginal, and none of the 44 correlations tested (11 compounds × 4 attributes) would survive a Bonferroni correction for multiple comparisons. Although nonanal showed a nominally significant correlation only with flavor, it also showed non-significant positive trends with appearance and taste. Together with its detection in all samples, this finding suggests that lipid oxidation-derived aldehydes likely contribute to the sensory characteristics of braised chicken feet.

**Fig. 7:**
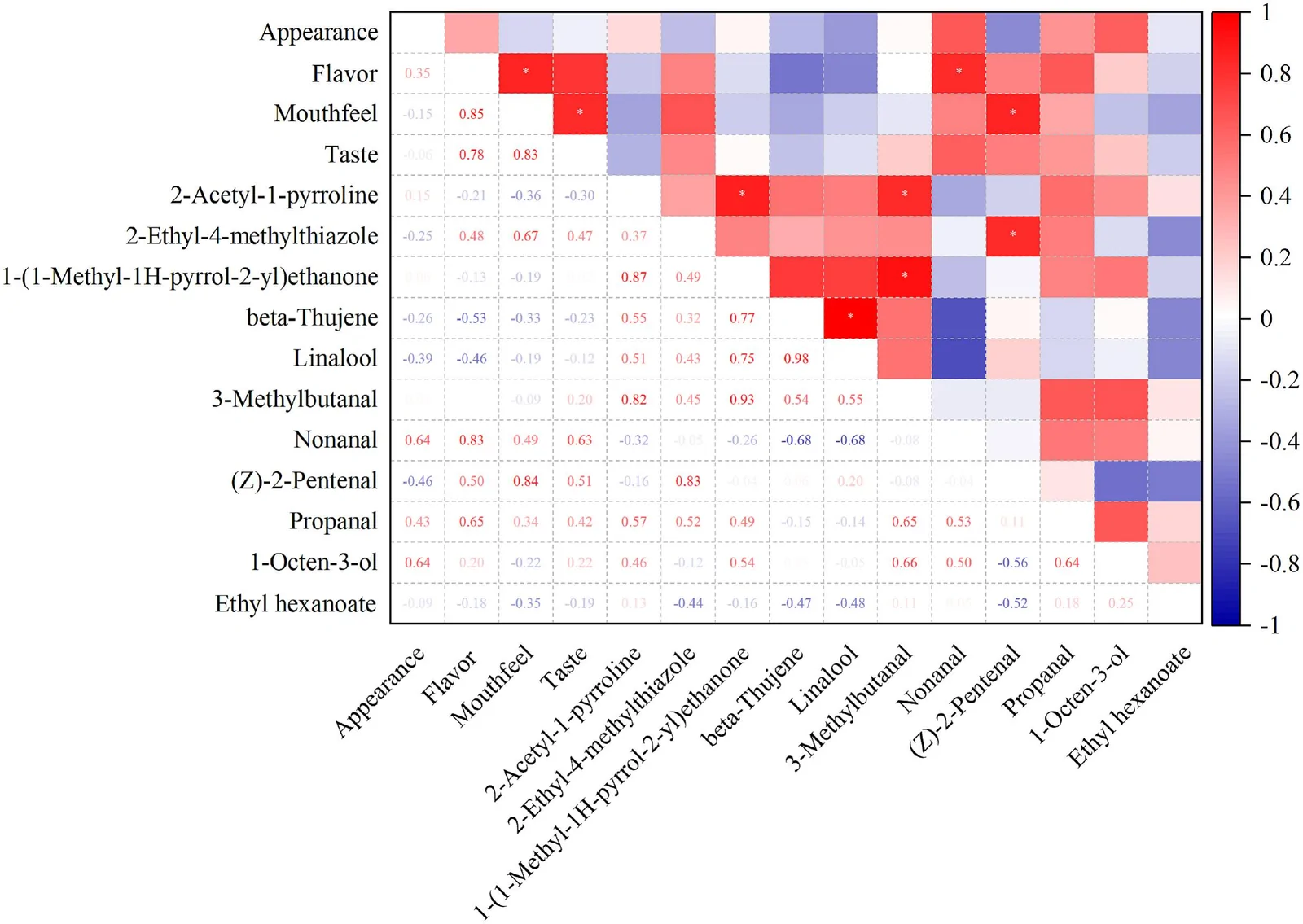
Correlation heatmap between sensory attributes and candidate aroma-active compounds in braised chicken feet.

Mouthfeel showed a nominally significant positive correlation only with (Z)-2-pentenal (*r* = 0.84) and a non-significant positive trend with 2-ethyl-4-methylthiazole (*r* = 0.67), indicating that lipid oxidation-derived compounds may play a role in mouthfeel perception, with Maillard reaction-derived heterocyclic compounds also potentially making a contribution. Taste showed a non-significant positive trend with nonanal (*r* = 0.63), suggesting that lipid oxidation-derived aldehydes may contribute to taste-associated sensory perception. Flavor showed a nominally significant positive correlation with nonanal (*r* = 0.83) and a non-significant positive trend with propanal (*r* = 0.65). Notably, 2-acetyl-1-pyrroline showed a weak negative correlation with flavor (*r* = −0.21). Although this correlation was not statistically significant, it may suggest that high levels of heterocyclic compounds could adversely affect overall flavor acceptability. The appearance attribute did not show a significant correlation with any of the identified compounds, and showed only a non-significant positive trend with nonanal and 1-octen-3-ol (*r* = 0.64 for both).

Taken together, the correlation analysis revealed that the compounds with nominal associations with sensory attributes were all aldehydes, corresponding to mouthfeel and flavor. These findings are consistent with a potential role of lipid oxidation in shaping the sensory profile of braised chicken feet. Although the correlations between Maillard reaction-derived compounds and sensory attributes were not statistically significant, several compounds showed non-significant trends that may warrant further investigation. Future studies incorporating larger sample sizes and sensory recombination experiments are needed to further clarify the sensory contributions of these compounds.

#### 3.6.2 Integration of aroma formation pathways

Taken together, the combined evidence from volatile profiling, multivariate analysis, and sensory correlation analysis reveals that the aroma profile of braised chicken feet is proposed to arise from three interconnected chemical pathways (Fig. 8) (Cheng et al., 2022; Fu et al., 2022), each contributing distinct aroma characteristics.

**Fig. 8:**
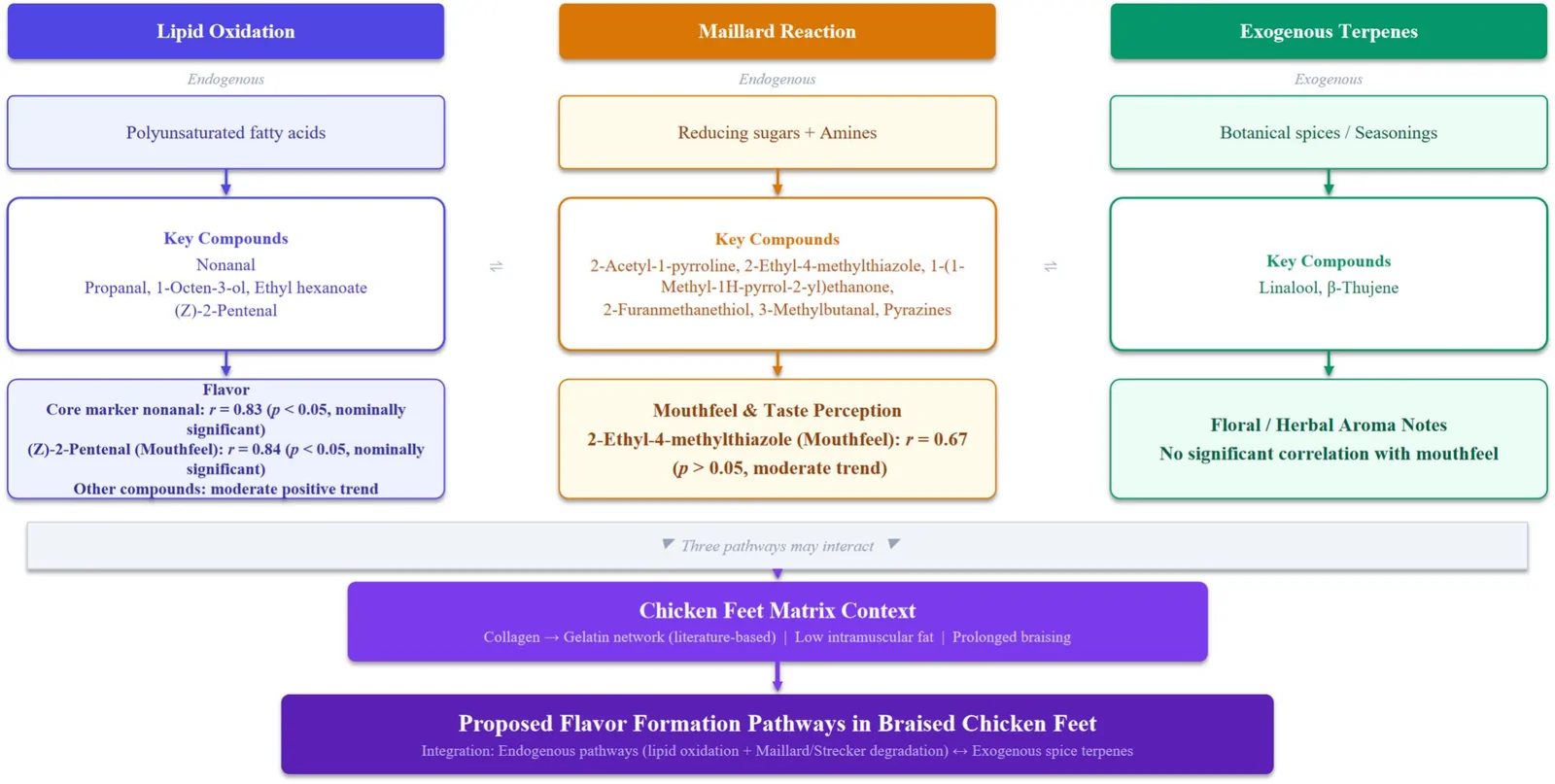
Schematic diagram of the proposed flavor formation pathways in braised chicken feet.

The endogenous lipid oxidation and Maillard reaction pathways likely provide a common aroma foundation (Liu et al., 2020; Zhang, Li, et al., 2025) across all samples, regardless of formulation. Lipid oxidation of endogenous polyunsaturated fatty acids generates a wide range of aldehydes, alcohols, and esters, including nonanal, propanal, (Z)-2-pentenal, 1-octen-3-ol, and ethyl hexanoate. These compounds collectively contribute fatty, green, fresh, and mushroom-like aroma characteristics that form the fundamental aroma background detected in all samples. Among these compounds, nonanal, a representative lipid oxidation product, exhibited a nominally significant positive correlation with flavor (*r* = 0.83, *P* < 0.05), whereas (Z)-2-pentenal also showed a nominally significant positive correlation with mouthfeel (*r* = 0.84), and the remaining lipid-derived compounds exhibited non-significant positive trends with sensory attributes. These findings support the contribution of lipid oxidation-derived compounds as a common background component of the overall aroma profile, even in a matrix with low intramuscular fat content.

The Maillard reaction pathway, arising from thermal interactions among reducing sugars, amino compounds, and free peptides released during cooking, generates a range of heterocyclic compounds and branched-chain aldehyde markers, including 2-acetyl-1-pyrroline, 2-ethyl-4-methylthiazole, 1-(1-methyl-1H-pyrrol-2-yl)ethanone, 2-furanmethanethiol, 3-methylbutanal, and various pyrazines (Cai et al., 2024; Dach & Schieberle, 2021; Lee et al., 2024). These compounds showed strong discriminatory potential among the samples, with substantial differences in relative abundance across the samples. Notably, 2-acetyl-1-pyrroline and 2-ethyl-4-methylthiazole, together with the spice-derived terpene linalool, exhibited the highest VIP scores in the OPLS-DA model. In terms of sensory associations, none of the Maillard reaction-derived compounds showed a statistically significant correlation with the sensory attributes; 2-ethyl-4-methylthiazole exhibited a non-significant positive trend with mouthfeel (*r* = 0.67, *P* > 0.05). These findings suggest that Maillard-derived compounds may influence other sensory attributes through potential cross-modal interactions, although this requires further investigation.

Complementing these endogenous reaction pathways, spice-derived terpenes (Liu et al., 2023; Sun et al., 2023) likely provide an additional source of aroma variation that may contribute to sample-specific flavor characteristics. Linalool and β-thujene were representative compounds in this category, showing the highest relative abundances and ROAV scores in the LJ and ZY samples, whereas substantially lower levels were observed in the other samples. Since terpenes are primarily introduced through spice blends rather than generated from the chicken matrix, their distribution likely reflects differences in spice composition and usage. In terms of odor character, terpenes primarily contribute floral, herbal, and spicy aroma notes and showed no significant correlation with mouthfeel. The variation in terpene abundance among the six samples supports a flavor differentiation pattern associated with spice intensity. In spice-rich samples, terpene-derived herbal and spicy notes may interact with or partially mask the subtle aroma contributions of lipid-derived aldehydes, whereas lipid oxidation products, particularly nonanal, remain dominant in milder samples (Yao, Huang, et al., 2024; Zhao et al., 2024). Further targeted sensory recombination experiments are needed to determine whether terpenes interact synergistically with Maillard-derived compounds to enhance roasted aroma characteristics or primarily contribute independent spice-related notes.

These three proposed pathways may converge and interact within the complex matrix of braised chicken feet. During prolonged braising, collagen in chicken feet is converted into a gelatinous network, while the inherently low intramuscular fat content may further modulate the release and perception of volatile compounds (Liu et al., 2020). Ultimately, the integration of endogenous pathways (lipid oxidation and Maillard reaction/Strecker degradation) with exogenous spice-derived terpenes may contribute to the overall flavor architecture of braised chicken feet. These three pathways thus remain hypotheses rather than established mechanisms.

## 4 Conclusions

This study systematically characterized the flavor chemistry and quality attributes of commercial braised chicken feet using GC-IMS, electronic nose analysis, sensory evaluation, and multivariate statistical analysis of six samples, including one laboratory-prepared sample and five commercial samples. Among the 81 identified volatile compounds belonging to 10 chemical classes, 12 candidate aroma-active compounds (ROAV ≥1) enabled clear discrimination among the six samples in the OPLS-DA model (R^2^Y = 0.996, Q^2^ = 0.991), with 2-acetyl-1-pyrroline, 2-ethyl-4-methylthiazole, and linalool identified as the three compounds with the highest VIP scores. Nonanal showed a nominally significant positive correlation with flavor (*r* = 0.83, *P* < 0.05), whereas its correlations with appearance and taste were non-significant. The laboratory-prepared sample ZZ received the highest scores for flavor, mouthfeel, and taste, although only taste differed significantly from all commercial samples. The flavor profile of braised chicken feet is proposed to arise from three interacting pathways: a common aroma background is likely provided by lipid oxidation, the major sample-to-sample differentiation is likely attributable to Maillard reaction/Strecker degradation products, and exogenous floral, herbal, and spicy odor notes are likely contributed by spice-derived terpenes. In addition, the collagen-rich matrix of chicken feet may influence the release of volatile compounds and sensory perception during prolonged braising. Because the six samples were each represented by a single production batch, the present findings are exploratory and should be confirmed by replication across independent production batches. These findings and the identified candidate aroma markers of flavor formation provide a scientific basis for understanding flavor formation processes, quality control, and product development in the braised poultry industry.

## Author contribution

Z.Z., writing—review and editing, methodology, and conceptualization; H.X., writing—review and editing, methodology, and conceptualization; Y.A., formal analysis; Z.M., M.L., formal analysis, validation, and supervision; L.C. (Lishui Chen), validation and supervision.

## CRediT authorship contribution statement

**Zijing Zhang:** Writing – review & editing, Writing – original draft, Conceptualization. **Haoyang Xie:** Formal analysis, Conceptualization. **Yongqiang An:** Formal analysis. **Zihao Meng:** Formal analysis. **Mengjie Li:** Formal analysis. **Longfei Wang:** Formal analysis. **Lishui Chen:** Writing – review & editing.

## Funding

This research was supported by the Henan Province Major Science and Technology Special Fund (No. 255101610001).

## Declaration of competing interest

The authors declare that they have no known competing financial interests or personal relationships that could have appeared to influence the work reported in this paper.

## Data Availability

Data will be made available on request.
