# Supplementary material for: Comparative characterization of volatile flavor profiles and quality attributes of laboratory-prepared and commercial braised chicken feet using a multi-analytical approach
Source: Food Chem X. 2026 Oct 26;40:104683. doi: 10.1016/j.fochx.2026.104683 (PMC13738294; doi:10.1016/j.fochx.2026.104683)
Supplement: Supplementary file 1 [file mmc1.docx]

**Supplementary material:**

FIGURE S1. Principal component analysis (PCA) score plot based on all 81 detected volatile compounds in the six braised chicken feet samples.

FIGURE S2. Orthogonal partial least squares discriminant analysis (OPLS-DA) score plot based on all 81 detected volatile compounds in the six braised chicken feet samples (R^2^Y = 0.998, Q^2^ = 0.997).

FIGURE S3. Permutation test (200 permutations) for the OPLS-DA model based on all 81 detected volatile compounds (intercepts: R^2^ = 0.181, Q^2^ = −0.843).

FIGURE S4. Variable importance in projection (VIP) scores from the OPLS-DA model based on all 81 detected volatile compounds.
